# Are Maternal Dietary Patterns During Pregnancy Associated with the Risk of Gestational Diabetes Mellitus? A Systematic Review of Observational Studies

**DOI:** 10.3390/nu16213632

**Published:** 2024-10-25

**Authors:** Charikleia Kyrkou, Apostolos P. Athanasiadis, Michael Chourdakis, Stefania Kada, Costas G. Biliaderis, Georgios Menexes, Alexandra-Maria Michaelidou

**Affiliations:** 1 Department of Food Science and Technology, School of Agriculture, Faculty of Agriculture, Forestry and Natural Environment, Aristotle University of Thessaloniki, 54124 Thessaloniki, Greece; ckyrkou@hotmail.gr (C.K.); biliader@agro.auth.gr (C.G.B.); 2 3rd Department of Obstetrics and Gynecology, School of Medicine, Faculty of Health Sciences, Aristotle University of Thessaloniki, 54124 Thessaloniki, Greece; apostolos3435@gmail.com; 3 Laboratory of Hygiene, Social & Preventive Medicine and Medical Statistics, School of Medicine, Faculty of Health Sciences, Aristotle University of Thessaloniki, 54124 Thessaloniki, Greece; mhourd@gapps.auth.gr (M.C.); stefania.kada@gmail.com (S.K.); 4 Department of Field Crops and Ecology, School of Agriculture, Faculty of Agriculture, Forestry and Natural Environment, Aristotle University of Thessaloniki, 54124 Thessaloniki, Greece; gmenexes@agro.auth.gr

**Keywords:** lifestyle factors, metabolic health, a posteriori, pregnancy complications, eating habits, unhealthy, western diet, prudent diet, nutritional quality

## Abstract

Background/Objectives: Maternal nutritional status is a “key” contributor to Gestational Diabetes Mellitus (GDM). However, the role of maternal dietary patterns (DPs) during pregnancy remains poorly understood. Thus, we conducted a systematic review to assess associations between “a posteriori-derived” DPs and GDM. Methods: A systematic search was conducted in PubMed, ScienceDirect, Web of Science, and Scopus for cohort, cross-sectional, and case–control studies published until June 2024. A total of twenty-eight studies involving 39,735 pregnant women were included, and their quality was evaluated by the Newcastle–Ottawa Scale. The 91 identified DPs were classified into four categories: “Westernized”, “Nutritious”, “Plant-based”, and “Miscellaneous”. Results: Our findings do not reveal definitive associations between maternal DPs during pregnancy and GDM risk. Notably, “Westernized” DPs tended to be associated with an increased risk. However, a very small portion of patterns within this category exhibited protective associations. Conversely, “Nutritious” and “Plant-based” appear beneficial for GDM prevention in specific populations. The “Miscellaneous” category presented an almost equal distribution of DPs with both detrimental and protective associations, pinpointing the absence of a clear directional trend regarding GDM risk. Conclusions: The heterogeneity in findings can be attributed to geographic and sociocultural variations and methodological differences across studies. Thus, there is a need for more standardized research methodologies to provide more precise insights that will ultimately help develop effective and tailored dietary guidelines for GDM prevention.

## 1. Introduction

Gestational Diabetes Mellitus (GDM) is a metabolic condition defined by varying levels of glucose intolerance during gestation in women with normal pre-pregnancy glucose metabolism [1]. Although it is a complex, multifactorial disorder [2,3], it is widely accepted that GDM is primarily triggered, in most cases, by pancreatic β-cell dysfunction in the presence of chronic insulin resistance [4,5]. 

As reported by the 10th edition of the International Diabetes Federation Diabetes Atlas, in 2021, over 21 million women experienced some form of hyperglycaemia during pregnancy, with GDM accounting for 80% of these cases [6]. This number is anticipated to multiply, mirroring the dramatic worldwide increase in obesity rates among women of reproductive age [7,8]. 

Alarmingly, this metabolic disorder not only leads to numerous adverse obstetric and fetal complications but also poses long-term risks extending beyond pregnancy and the neonatal period [4,6,9,10,11]. Even though GDM typically resolves postpartum, women with a history of GDM face a 50% higher risk of developing diabetes mellitus (DM) and a 63% elevated risk of cardiovascular diseases later in life, mainly due to the chronic low-grade inflammation associated with insulin resistance [2,4,5,12]. Furthermore, the in utero overnutrition associated with GDM can contribute to an intergenerational cycle of obesity and DM through epigenetic changes, amplifying health and economic burdens [4,6,13,14]. Addressing GDM is, therefore, a critical global health priority. 

Among the various risk factors for GDM, the maternal intake of multiple nutrients, bioactive compounds, and foods plays a crucial role in GDM prevalence, as highlighted by numerous studies [1,14,15,16,17]. As such, a dietary pattern high in red meat, sweets, and highly processed foods is anticipated to heighten the risk of GDM due to the simultaneous over-intake of nutrients—such as saturated fat, cholesterol, branched-chain amino acids, sugars, and heme iron—that foment insulin resistance, oxidative stress, and low-grade inflammation [14,15,16,17]. Conversely, it is generally believed that diets rich in vegetables, whole grains, and other plant-based foods offer protective effects against GDM mainly due to their capability to induce satiety and improve glycaemic response [15,17,18]. However, the findings are quite inconsistent when the maternal diet is investigated from the “whole-diet approach”, the most promising methodology for assessing the potential association between diet and health outcomes [19,20,21]. For example, there are studies describing Western dietary patterns exhibiting positive [22,23], null [24,25], or even negative [26] associations with the manifestation of GDM, highlighting not only the complex interactions—both synergistic and antagonistic—between different nutrients and foods but also the potential effect of several additional parameters.

A limited number of systematic reviews have also tried to summarize the existing evidence and unravel the exact role of maternal dietary patterns on GDM [27,28,29]. However, no solid conclusions were extracted. In one of the first systematic reviews in this field, Kibret et al. (2018) [27] reported that a higher adherence to healthy dietary patterns reduced risks of developing GDM, while no links between “Western” dietary patterns and odds of GDM were described. More recently, Haghighatdoost et al. (2022) reported conflicting results on the association between “healthy” and “unhealthy” dietary patterns and GDM risk [29]. However, this evidence [27,28,29] has primarily relied on broad categorizations of dietary patterns (e.g., “healthy” vs. “unhealthy”), which may not fully capture the complexity of dietary influences on GDM. Moreover, the methodologies followed for dietary pattern extraction are not consistently taken into consideration. It is well known that in the “whole-diet approach”, the methodologies toward identifying dietary patterns can generally be grouped as “a priori” and “a posteriori” dietary pattern analysis [19,20,30]. While “a-priori” and “a-posteriori” methods offer valuable insights, their distinct theoretical foundations lead to different dietary patterns extracted. The “a-priori” approach constructs dietary patterns based on existing knowledge of a “high-quality” diet [20,30]. In contrast, the “a-posteriori” approach derives patterns directly from population data, incorporating personalized characteristics such as behavioural and cultural factors, thereby providing a more holistic understanding of diet–health associations [20]. 

Meanwhile, despite the increasing interest in this research field, the most updated observation data from the last four years has not yet been evaluated in the context of a systematic review. Therefore, to stay aligned with the rapidly evolving research, this systematic review aims to synthesize the latest observational data on dietary patterns and their association with GDM risk during pregnancy. In contrast to previous published systematic reviews [27,28,29], we exclusively focused on “a posteriori” dietary patterns. Moreover, we have incorporated only studies investigating maternal dietary patterns during pregnancy, considering the distinct physiological, metabolic, and behavioural adaptations that occur during this period. 

## 2. Materials and Methods

**Figure 1:**
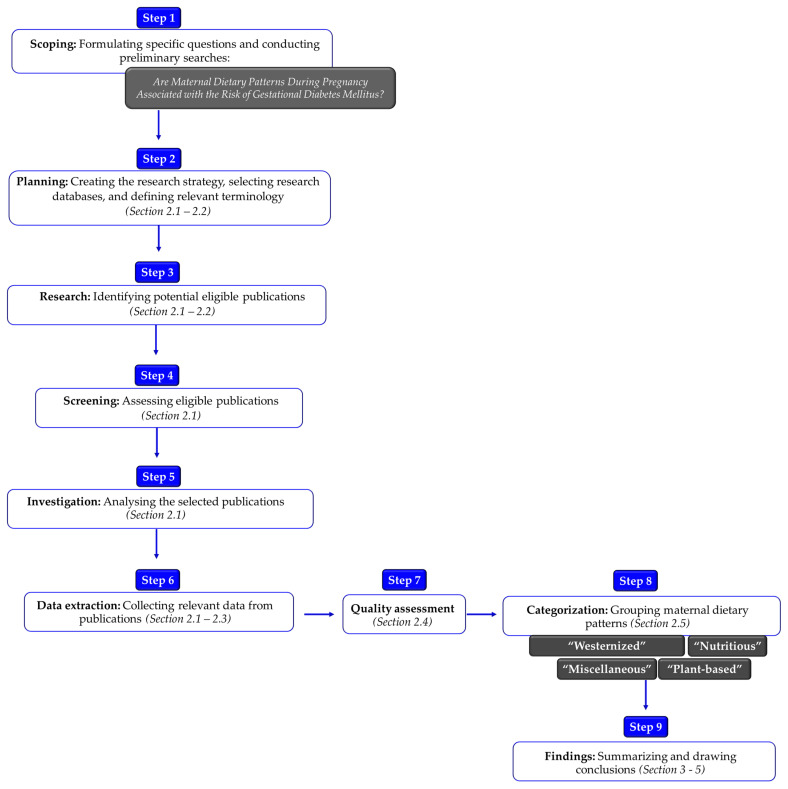
The framework of the methodological design followed in the current systematic review.

All authors developed and unanimously agreed upon a detailed protocol to carry out this systematic review (Figure 1). 

The review adhered to all the essential steps outlined by the Preferred Reporting Items for Systematic Reviews and Meta-Analyses (PRISMA) guidelines [31]. The study protocol has been registered in the Open Science Framework (OSF) (osf.io/at3ue).

### 2.1. Research Strategy

Table 1 lists the search strategy terms used for each electronic database (PubMed, Science Direct, Web of Science, Scopus). We evaluated original research articles identified in the initial screening based on predetermined exclusion and inclusion criteria (see Section 2.2). When it was not possible to determine an article’s eligibility from the title and abstract alone, we conducted a thorough review of the full text. Additionally, we manually checked the reference lists of the publications that met the eligibility criteria and previous reviews on the subject. We also utilized the “cited by” feature of the Google Scholar search engine to uncover further publications that the initial search might have missed, aiming to gather as many studies as possible. 

Two independent reviewers (C.K. and S.K.) meticulously scrutinized each of the selected electronic databases for eligible articles published until 30 January 2024, while a third (A.-M.M.) and a fourth evaluator (A.P.A.) rigorously deliberated and resolved any discrepancies until a consensus was achieved. 

To ensure no additional publications emerged during the evaluation process, a supplementary search was conducted to capture any papers published from 1 March to 31 July 2024.

### 2.2. Inclusion and Exclusion Criteria

For inclusion in the review, articles needed to meet the following criteria: (a) be original research that explores the potential link between maternal “a posteriori” dietary patterns during gestation and GDM risk; (b) employ an observational study design, such as cohort, cross-sectional, or case–control; (c) be published in peer-reviewed journals in English; and (d) involve healthy adult pregnant women carrying a single fetus. 

We excluded articles if (a) they discussed the aforementioned associations on a theoretical basis (for instance, systematic or narrative reviews, editorials, and letters to editors); (b) dietary assessments were performed after the GDM diagnosis or included women already diagnosed with DM (type 1 or 2); (c) dietary data were collected before pregnancy; (d) the full text was unavailable; and (e) the association between maternal diet and GDM risk was not clearly delineated.

### 2.3. Data Extraction

For each article selected for inclusion, we conducted a thorough review and extracted the following information:

- Study details, including author names, year of publication, and study design.
- Population details such as the total number of participants, the percentage (%) of GDM cases, and the location of participant recruitment.
- Details on exposure assessment, including the dietary assessment method, the period (gestational week—trimester) over which it was assessed, and the statistical analysis used to extract dietary patterns.
- Information on outcome assessment detailing the method used for GDM screening and the diagnostic criteria.
- Results, including the dietary patterns identified, the associations between these patterns and GDM, and any covariates or confounding factors considered.

### 2.4. Quality Assessment

To evaluate the quality of the case–control and cohort studies, we applied the Newcastle–Ottawa Scale (NOS) [32]. For cross-sectional studies, we used the NOS modifications developed by Herzog et al. (2013) [33]. The quality of each study was assessed across three key categories/domains: selection, comparability, and outcome (or exposure for case–control studies). This assessment resulted in a summary score for each study, with possible scores ranging from zero to nine stars for cohort and case–control studies [32] and zero to ten stars for cross-sectional studies [33,34]. Details of the NOS checklists (items/questions for each category) are available in Supplementary Materials SI (Tables SI.1–SI.3). Studies were categorized as low quality (≤3 stars), medium quality (4–6 stars), or high quality (≥7 stars). The quality assessment was performed independently by two reviewers, C.K. and A.-M.M., with any disagreements resolved through detailed discussions with a third evaluator (G.M.).

### 2.5. Categorization of Maternal Dietary Patterns

To thoroughly investigate the potential association between maternal “a-posteriori” dietary patterns during gestation and GDM risk, we categorized the maternal dietary patterns derived from the 28 surveys under examination into four distinct classes (“Westernized “, “Nutritious”, “Plant-based”, and “Miscellaneous”). This categorization was based on the similarities in the combination of commonly consumed food groups [14,27,35] and was independently conducted by three reviewers (C.K., A.-M.M, and M.C.). Additionally, where possible, we considered the designations provided in the original articles to ensure consistency and accuracy.

- “Westernized”: This umbrella term encompasses dietary patterns designated as “Western”, “Μeat-based”, “Sweet foods”, “Processed”, “Junk”, etc. The commonality among these patterns lies in their promotion of energy-dense, nutrient-poor foods [14,16]. Consistent with definitions and descriptions found in previous research [14,28], these dietary patterns are characterized by the excessive intake of refined cereals, red and processed meat, fast foods, sweets, beverages, and other energy-dense foods and limited consumption of fruits, vegetables, whole grains, and nuts. These foods are typically rich in total and saturated fat and have an elevated glycaemic index, contributing to an unbalanced dietary profile [14,16,29,36].
- “Plant-based”: A general term that refers to dietary habits focusing on primarily consuming vegetables, fruits, and other plant-origin foods, such as cereals and pulses, while reducing meat consumption and other animal-based products [37]. These food groups are essential sources of fibre, vitamins, minerals, and phytochemical compounds [29,35,37].
- “Nutritious”: This is also an umbrella term for “Nutritious”, “Prudent”, “Health-conscious”, and other dietary patterns that share several key characteristics focused on promoting overall health [28,29]. These dietary patterns are defined by high consumption of whole-grain cereals, fruits, vegetables, white meat, and fish. Given that dairy products are essential for meeting increased maternal calcium requirements during pregnancy [35], we also incorporated patterns characterized by a high intake of either low- or full-fat dairy products. Furthermore, these patterns prioritize balanced nutrition, ensuring the adequate intake of fibre, vital micronutrients, and bioactive compounds and the low consumption of added sugars and total fat [38,39,40].
- “Miscellaneous”: These patterns feature a unique combination of specific food items—i.e., the inclusion of energy-dense, nutrient-poor foods alongside more traditional nutrient-rich staples—reflecting a blend of conventional and contemporary dietary choices shaped by the influences of westernization/globalization, cultural heritage, socioeconomic status, and individual preferences [41]. For example, red meat and junk food are combined with fruits, vegetables, poultry or fish, and traditional cereal-based products [42]. Thus, although systematically identified in nutritional research [41,42,43], these dietary patterns could not strictly fit into one of the previously defined categories, forming a diverse and heterogeneous group collectively referred to as “miscellaneous”.

### 2.6. Analysis of Stratified Data

To identify potential sources of heterogeneity among the reviewed studies and comprehensively evaluate the possible associations between maternal “a-posteriori” dietary patterns during pregnancy and the risk of GDM, we conducted a stratified analysis within each dietary pattern category (“Westernized”, “Nutritious”, “Plant-based”, and “Miscellaneous”). This analysis entailed stratifying the data by study design (cohort, cross-sectional, and case–control), study location (developed nations versus less developed nations/emerging economies), dietary pattern extraction methodology (Factor Analysis [FA], Principal Component Analysis [PCA], K-means cluster analysis, and Reduced Rank Regression [RRR]), and the trimester of maternal dietary assessment (first, first and second, second, second and third, third trimester).

## 3. Results

### 3.1. Study Selection

**Figure 2:**
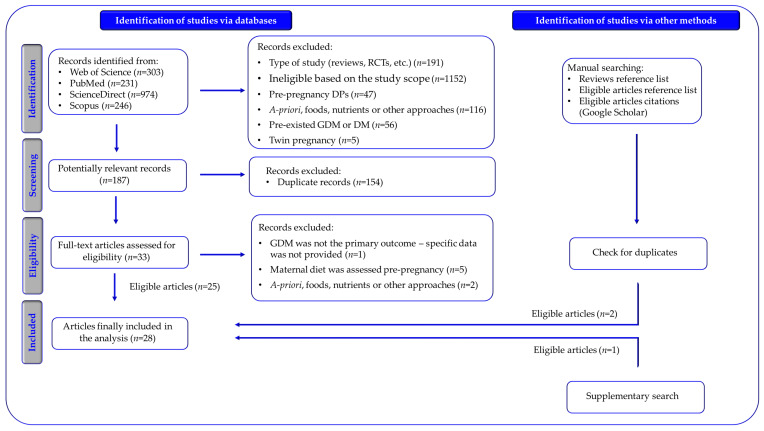
Schematic representation of the study selection procedure followed in the current systematic review (adopted from the PRISMA 2020 Flow Diagram [31]). RCTs: Randomized Controlled Trials; DPs: Dietary Patterns; GDM: Gestational Diabetes Mellitus; DM: Diabetes Mellitus.

Figure 2 depicts the comprehensive methodology for selecting studies in this systematic review. Initially, the literature search yielded 1754 articles: 303 from the Web of Science, 231 from PubMed, 974 from ScienceDirect, and 246 from Scopus. After screening the titles and abstracts, 1567 articles were excluded for not meeting our inclusion criteria. From the remaining 187 full-text articles that were reviewed for eligibility articles, 154 duplicate records were removed. Subsequently, an in-depth review of the full texts resulted in the exclusion of five additional articles that did not meet the inclusion criteria. 

Initially, 25 articles were deemed eligible for inclusion. An additional three studies were identified through Google Scholar’s “cited by” feature, relevant reference lists, and supplementary searches, resulting in a final total of twenty-eight included studies.

### 3.2. Study Characteristics

**Figure 3:**
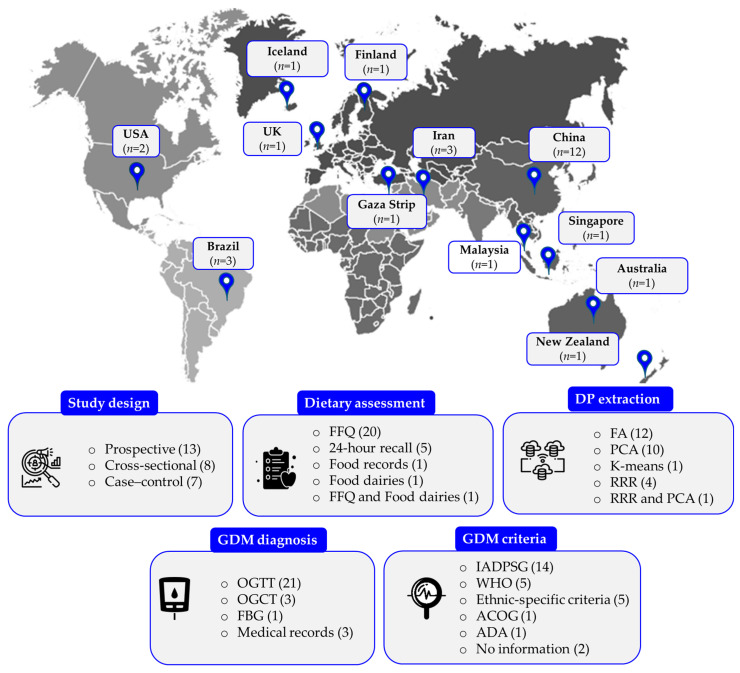
Overview of the study characteristics (*n* = 28). *n*: number of studies. The numbers in parenthesis indicate how many of the 28 studies reported/presented that specific characteristic. ACOG: American College of Obstetricians and Gynecologists; ADA: American Diabetes Association; DP: Dietary patterns; FA: factor analysis; FBG: Fasting Blood Glucose; FFQ: Food Frequency Questionnaire; IADPSG: International Association of Diabetes and Pregnancy Study Groups; OGCT: Oral Glucose Challenge Test; OGTT: Oral Glucose Tolerance Test; PCA: Principal Component Analysis; RRR: Reduced Rank Regression, WHO: World Health Organization.

Figure 3 provides an overview of the geographical distribution and methodological details of 28 studies evaluated in the current systematic review. 

The included studies consisted of thirteen cohort studies [23,24,26,44,45,46,47,48,49,50,51,52,53], eight cross-sectional studies [7,22,25,54,55,56,57,58], and seven case–control studies [8,59,60,61,62,63,64] (Figure 3), with sample sizes ranging from 168 [46] to 9556 [8] participants (Table 2). The overall sample size was 39,735 women. The studies were conducted in 12 countries. Significantly, China was a major contributor to this body of research, highlighting a low geographic diversity of the data. Among the 28 studies reviewed, which range from 2007 [44] to 2024 [58,64], more than one-third (11 out of 28, or 36%) [8,52,53,56,57,58,60,61,62,63,64] were published after March 2020. This indicates that our analysis includes the most up-to-date research in this field.

The follow-up durations in the thirteen cohort studies varied from 2 [49,50] to 23 weeks [23], although three studies did not provide specific follow-up periods [26,44,53]. De Seymour et al. (2022) [52] conducted follow-ups with participants every trimester, whereas Nascimento et al. (2016) [47] continued follow-ups until delivery. 

In most studies included in the review (*n* = 20), the Food Frequency Questionnaire (FFQ) was the primary tool for assessing diet (Figure 3). Of these, 15 studies confirmed that the FFQ had been previously validated [8,24,44,45,47,48,49,51,53,57,58,59,60,61,63] (Table 2). Additionally, two research groups, Flynn et al. (2016) [22] and Hu et al. (2019) [50], presented correlation analysis data to demonstrate that their FFQs accurately assessed maternal diet. Alternative dietary assessment methods included the 24 h recall method (24 hR) (*n* = 5), [7,23,25,54,55], the four-day food records (FRs) (*n* = 1) [46], and the food diaries (*n* = 1) (FDs) [56] (Table 2). Twenty-three studies utilized data reduction techniques to create dietary patterns, including FA in twelve studies [8,22,24,44,45,47,48,50,52,54,61,64], PCA in ten studies [7,23,26,46,49,51,56,58,59,60], and K-means cluster analysis in one study [57]. Additionally, four surveys proposed the RRR [25,55,62,63] (Figure 3). Notably, Wang et al. (2023) employed both the PCA and RRR [53] (Table 2).

As far as GDM diagnosis is concerned, most studies (*n* = 21) employed the oral glucose tolerance test (OGTT) for screening [7,22,45,46,47,48,49,50,51,52,53,54,55,56,57,58,60,61,62,63,64] and applied the diagnostic criteria set by the International Association of Diabetes and Pregnancy Study Groups (IADPSG) [8,22,23,25,45,47,48,49,50,52,53,57,63,64]. Interestingly, only five applied the World Health Organization (WHO) cut points [7,46,54,55,61], while a number of studies adopted ethnic-specific criteria [26,51,56,58,62] (Figure 3). The reported prevalence of GDM among cross-sectional and cohort studies varied widely, ranging from 5% [26,44] to 28% [52] (Table 2), reflecting the differences in study populations and the diagnostic criteria employed.

Except for the studies conducted by Pajunen et al. [56] and Waheby et al. [61], nearly all research adjusted their findings for maternal age (Table 2). Additionally, most studies accounted for Body Mass Index (BMI) (*n* = 22) [8,22,23,25,26,44,45,47,48,49,50,52,53,54,56,57,58,59,60,62,63,64], maternal educational status (*n* = 18) [7,8,23,25,45,47,48,49,50,52,53,54,55,57,59,60,62,63], and the history of GDM or DM (*n* = 16) [7,8,23,44,45,47,48,49,51,53,54,55,57,59,61,63]. In contrast, fewer studies (*n* = 10) included adjustments for gestational weight gain (GWG) [8,25,46,49,55,59,60,61,62,63]. Other confounding factors considered by some studies included ethnicity, physical activity, socioeconomic status, energy intake, and reproductive factors such as parity and the number of deliveries (Table 2).

**Figure 4:**
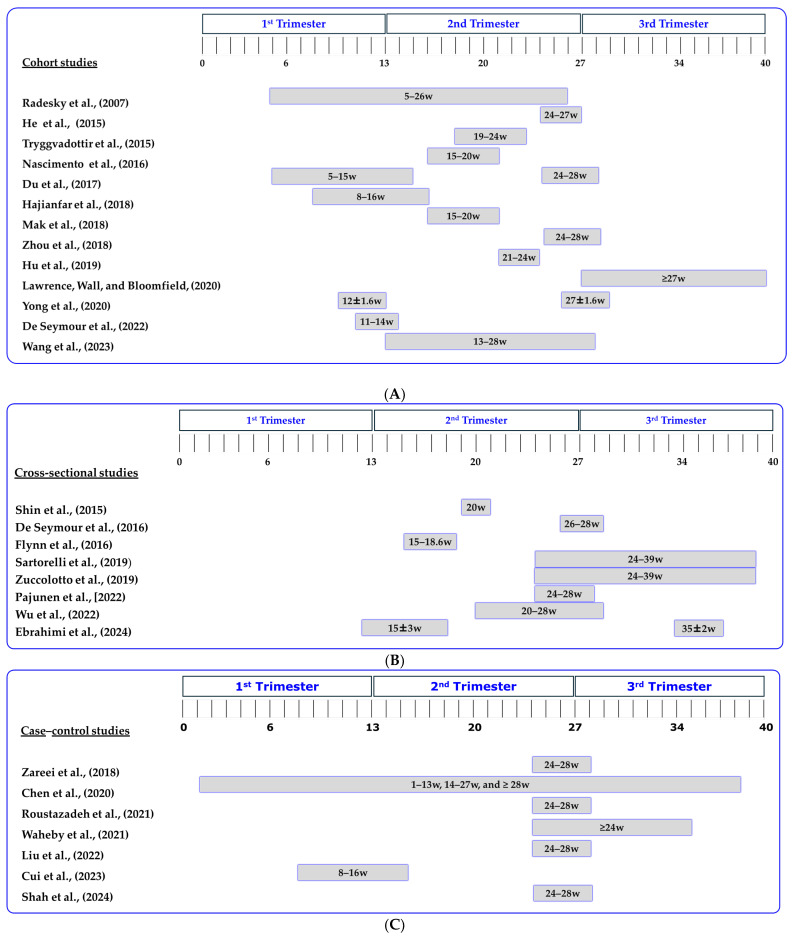
(**A**) Dietary assessment period across the 13 cohort studies under investigation. w: weeks of gestation [23,24,26,44,45,46,47,48,49,50,51,52,53]. (**B**) Dietary assessment period across the 8 cross-sectional studies under investigation. w: weeks of gestation [7,22,25,54,55,56,57,58]. (**C**) Dietary assessment period across the 7 case–control studies under investigation. w: weeks of gestation [8,59,60,61,62,63,64].

In most cohort studies, researchers assessed maternal diet between the 5th and 28th weeks of gestation, as depicted in Figure 4A. However, some participants in the study by Lawrence, Wall, and Bloomfield (2020) [26] provided dietary data after the diagnosis of GDM. Similarly, all cross-sectional studies gathered dietary data between the 12th and 39th weeks of gestation, as shown in Figure 4B. Case–control studies, however, exhibited significant variations in the timing of nutritional assessments, as illustrated in Figure 4C.

### 3.3. Quality Assessment

Our assessment, utilizing the NOS checklist (Supplementary Material SI, Tables SI.1–SI.3), revealed that the quality scores of the evaluated surveys ranged from 5 (indicating a moderate degree of bias) to 9 (suggesting a low degree of bias). Consequently, all publications evaluated were categorized as either medium or high quality. Specifically, 85% (eleven out of thirteen) of the cohort studies, 75% (six out of eight) of the cross-sectional studies, and 71% (five out of seven) of the case–control studies achieved a high-quality rating (score ≥ seven stars). All the necessary details on the ratings of each survey are available in Supplementary Materials SII (Tables SII.1–SII.3).

**Figure 5:**
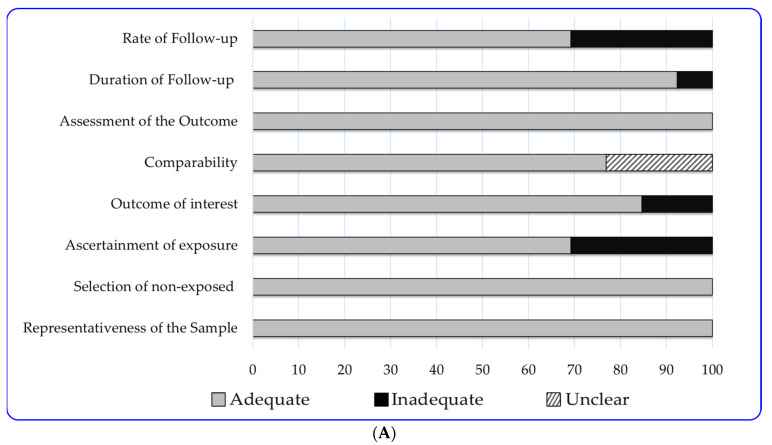

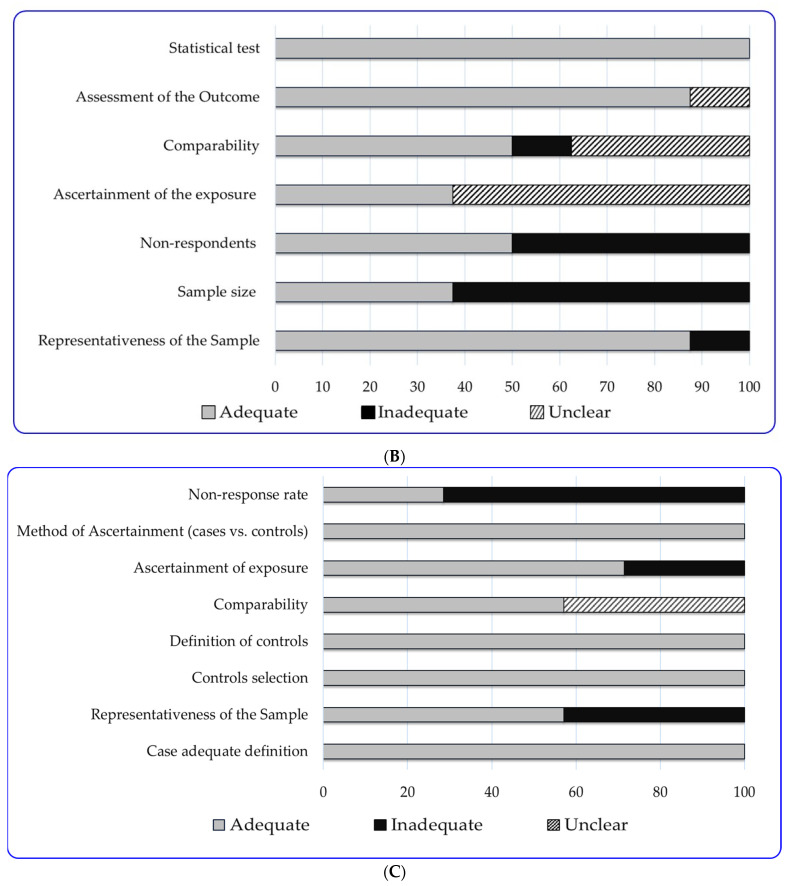
(**A**) Quality assessment of the 13 cohort studies evaluated. The bars represent the percentages of studies categorized as “Adequate”, “Inadequate”, and “Unclear” across each of the eight criteria that comprise the Newcastle–Ottawa score. A study was rated “Adequate” if it achieved the maximum score on a given criterion. Studies that received 0 stars were categorized as “Inadequate.” Studies that received a score of 1 out of 2 stars for the “Comparability” criterion were categorized as “Unclear”. (**B**) Quality assessment of the 8 cross-sectional studies evaluated. The bars represent the percentages of studies categorized as “Adequate”, “Inadequate”, and “Unclear” across each of the seven criteria that comprise the Newcastle–Ottawa score. A study was rated “Adequate” if it achieved the maximum score on a given item. Studies that received 0 stars were categorized as “Inadequate.” For items with a maximum score of 2 stars (assessment of the outcome, comparability, and ascertainment of the exposure), studies scoring 1 star were labelled as “Unclear”. (**C**) Quality assessment of the 7 case–control studies evaluated. The bars represent the percentages of studies categorized as “Adequate”, “Inadequate”, and “Unclear” across each of the eight criteria that comprise the Newcastle–Ottawa score. A study was rated “Adequate” if it achieved the maximum score on a given item. Studies that received 0 stars were categorized as “Inadequate.” Studies that received a score of 1 out of 2 stars for the “Comparability” criterion were categorized as “Unclear”.

As illustrated in Figure 5A (Supplementary Materials SII—Table SII.1), all cohort studies adequately addressed the items/questions related to “representativeness of the sample”, “selection of non-exposed”, and “assessment of the outcome”. Interestingly, although the majority (92%, 12 out 13) of these studies provide information on the “duration of follow-up”, 31% (*n* = 4) lack information on subjects lost to follow-up and were rated as “inadequate” in the “rate of follow-up” item (receiving 0 stars). Meanwhile, 31% of the cohort studies were rated as “inadequate” in “Ascertainment of exposure” because the dietary assessment tool was either not validated or relied on self-reporting. No studies were rated as “inadequate” for the “comparability” item since all were controlled for maternal age and BMI.

All cross-sectional studies (*n* = 8) received an “adequate” rating for the “Statistical test” item because the authors disclosed the statistical tests used to analyze potential associations between maternal dietary patterns and GDM, including confidence intervals (95% CI) and *p*-values (Figure 5B, Supplementary Materials SII—Table SII.2). However, 63% (five out of eight) of these studies did not justify their sample size, such as through an a-priori power analysis (or another method), or the sample size was not sufficiently large, resulting in an inadequate rating for the “sample size” item [33,34]. Additionally, although researchers described the dietary assessment tools used, in most cases (63%, five out of eight), no information was provided on their validity, which led to a rating of only one star in the “Ascertainment of the exposure” item.

In the case–control studies (Figure 5C, Supplementary Materials SII—Table SII.3), all reviewed studies (*n* = 7) satisfied the adequacy criteria for four of the eight NOS items. However, the majority (*n* = 5, 71%) did not provide specific information regarding the rate or characteristics of non-responders (“Non-response rate” item). In the remaining items, the proportion of studies rated as “adequate” varied from 57% (“Representativeness of the Sample” and “Comparability”) (four out of seven studies) to 71% (“Ascertainment of exposure”) (five out of seven).

### 3.4. Potential Associations Between Maternal Dietary Patterns and GDM

**Figure 6:**
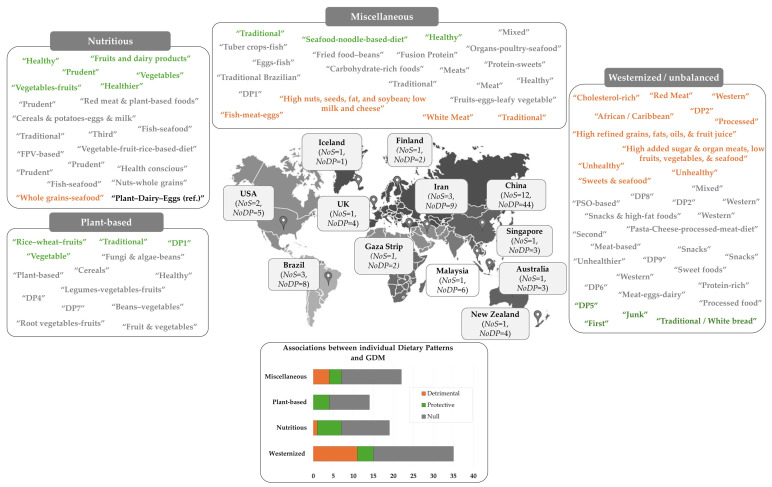
Overview of dietary patterns identified in the reviewed studies. Dietary pattern classifications are indicated in the labels surrounding the global map. Those marked in orange presented a detrimental link with GDM, those in green were protective against GDM, and those in grey showed no significant association with GDM risk. NoS = Number of Studies; NoDP= Number of Dietary Patterns.

Figure 6 illustrates a schematic layout of the dietary patterns (*n* = 91) identified across the studies examined, organized by country, and divided into four distinct categories: “Westernized”, “Plant-based”, “Nutritious”, and “Miscellaneous”. Dietary patterns in each study ranged from two to six. Table SIII.1 (Supplementary Materials SIII) provides the number and labels of dietary patterns identified across the 28 studies and a comprehensive summary of associations between maternal “a-posteriori” dietary patterns and GDM risk, focusing exclusively on the most adjusted models. In most cases, researchers designated the patterns identified based on the predominant food groups, such as “Beans–vegetables”, “Cereals”, and “Fruit and vegetables” (Figure 6—Table SIII.1). Additionally, twenty-seven were nominated as “traditional” [7,23,24,26,47,50], “healthy/-ier” or “health-conscious” [7,24,26,56,59,61], “unhealthy/-her” [56,59,61], “western” [23,24,44,47], “prudent” [23,44,45,46], and “mixed” [23,47]. Fourteen dietary patterns remained unlabelled (e.g., DP1, DP5, or First) [51,55,58,62]. In six instances, the labelling of patterns was based on nutrient content [26,45,48,50,60,63].

We deliberately excluded the “coffee” dietary pattern identified by Zuccolotto et al. (2019) from the classification, as it did not fit into any of the predefined categories due to its distinctive composition (Section 2.5) [7]. Additionally, the “DP3” dietary pattern identified by Liu et al. (2022) [62] was excluded from the categorization. This decision was made because the original study found that this pattern accounted for only a small percentage of variation (5.63%), rendering it insufficiently significant for further analysis.

✓ Westernized Dietary Patterns

**Figure 7:**
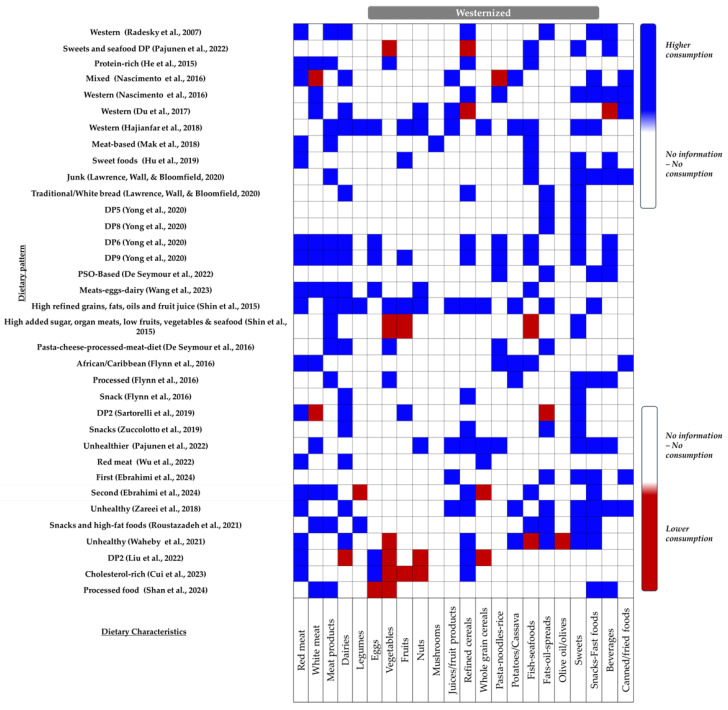
Combination of key food groups defining the “Westernized” dietary pattern. Blue indicates higher consumption, while red indicates lower consumption [7,22,23,24,25,26,44,45,47,48,50,51,52,53,54,55,56,57,58,59,60,61,62,63,64].

“Westernized” dietary patterns—characterized by the excessive intake of refined cereals, red and processed meat, fast foods, sweets, beverages, and other energy-dense foods and limited consumption of fruits, vegetables, whole grains, and nuts (Figure 7)—were identified in 25 publications (Table SIII.1). In five studies (i.e., six dietary patterns), high compared to low adherence was statistically significantly associated with an elevated GDM risk (one cross-sectional: [25]; four case–control: [59,61,62,63]). Meanwhile, both Du et al. (2017) [23] and Wu et al. (2022) [57] reached similar conclusions when “westernized” dietary patterns (“western” and “Red meat”, respectively) were compared with a “nutritious” pattern. 

Flynn et al. (2016) [22], in a cross-sectional survey among 1023 pregnant women in the UK, revealed that higher adherence (upper vs. lower quartile) to either the “processed” or the “African Caribbean” category was linked with an elevated GDM risk (OR: 2.05, 95% CI: 1.23–3.41, *p* = 0.022, and OR: 2.46, 95% CI: 1.41–4.31, *p* = 0.010, respectively). However, no significant associations were reported between the “snacks” dietary pattern and GDM risk (OR: 1.24, 95% CI: 0.76–2.01, *p =* 0.666) [22]. Such conflicted findings were also reported in the study of He et al. (2015) (“Sweets and seafood pattern”: positive associations, “protein-rich”: null associations) [45].

The lack of associations between maternal diet and GDM risk was similarly observed in twelve additional surveys (i.e., fourteen dietary patterns) (seven cohort: [24,44,47,48,50,52,53]; four cross-sectional: [7,54,55,56]; two case–control: [60,64]). 

Contradictory, three studies (four dietary patterns) suggested that “Westernized” dietary patterns may confer protection against GDM risk (two cohort: [26,51]; one cross-sectional: [58]). Nevertheless, as reported by both Yong et al. (2020) [51] and Lawrence, Wall, and Bloomfield (2020) [26], these associations were mainly driven by the specific characteristics of the study sample, such as their BMI or the timing of GDM diagnosis.

✓ Nutritious Dietary Patterns

**Figure 8:**
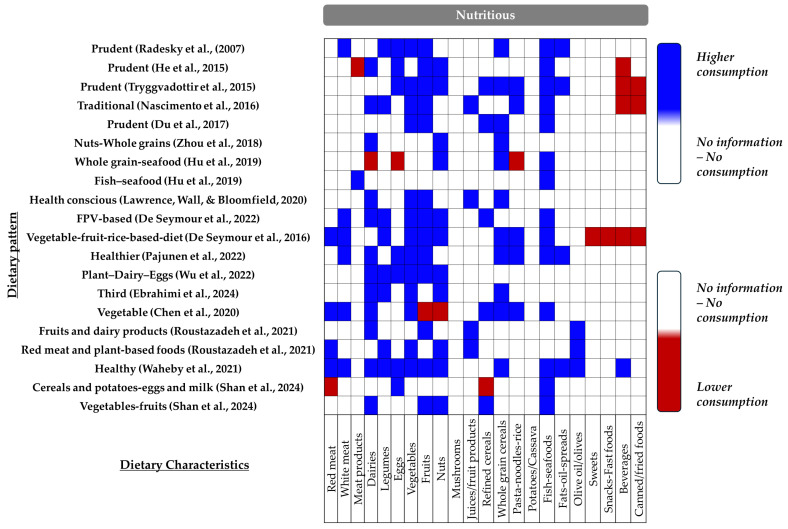
Combination of key food groups defining the “Nutritious” dietary patterns. Blue indicates higher consumption, while red indicates lower consumption [8,23,26,44,45,46,47,49,50,52,54,56,57,58,60,61,64].

Nutritious dietary patterns—including high intakes of vegetables, fruits, whole grains, lean proteins, and healthy fats (Figure 8)—were identified in seventeen studies (i.e., twenty dietary patterns) (Figure 6—Table SIII.1). Four studies found that higher adherence to “nutritious” reduced the incidence of GDM (one cohort: [46]; In total, one cross-sectional: [56]; two case–control: [8,61]). However, most studies (*n* = 9 studies) did not find evidence to support such effects (seven cohort: [23,26,44,45,47,49,52]; two cross-sectional: [54,58]). 

Shah et al. (2024) extracted two dietary patterns—“Cereals and potatoes-eggs and milk” and “Vegetable-fruits”—characterized by high consumption of nutritious foods (Figure 8). However, only higher factor scores (≥0.07 vs. <0.07) in the “Vegetables-Fruits” pattern were associated with a reduced risk of GDM (OR: 0.33; 95% CI: 0.15–0.74, *p* = 0.008) [64]. Similar findings were reported by Roustazadeh et al. (2021) (Table SIII.1) [60].

Unexpectedly, the Born in Shenyang Cohort Study, a prospective cohort study involving Chinese women, reported conflicting results [50]. Notably, higher adherence to the “Whole grain-seafood” dietary pattern was linked to a higher likelihood of being diagnosed with GDM (RR: 1.73, 95% CI: 1.10–2.74, *p* = 0.007), whereas no associations were observed for the “Fish-seafood” pattern [50]. The “Plant–dairy–eggs” DP, in the cross-sectional study conducted by Wu et al. (2022), was only used as a reference [57].

✓ Plant-based Dietary Patterns

**Figure 9:**
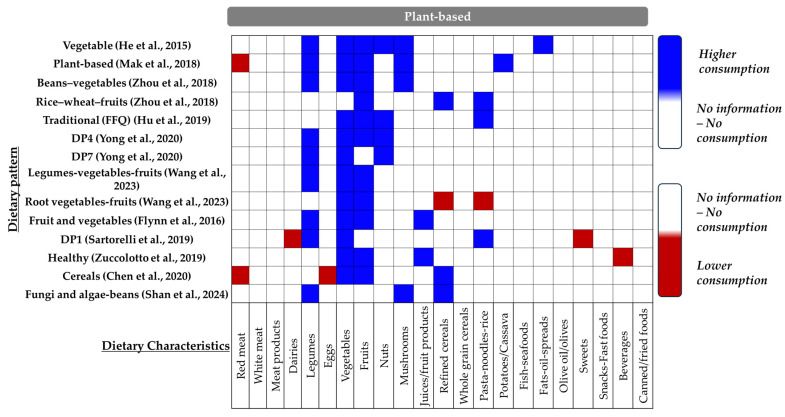
Combination of key food groups defining the “Plant-based dietary patterns”. Blue indicates higher consumption, while red indicates lower consumption [7,8,22,45,48,49,50,51,53,55,64].

Plant-based dietary patterns emphasizing vegetables, fruits, and other plant sources (Figure 9) were explored in 11 studies (14 dietary patterns) (Figure 6). Evidence from three research groups suggested that plant-based dietary patterns (*n* = 3) could significantly prevent this pregnancy complication (two cohort: [45,50]; one cross-sectional: [55]) (Figure 6—Table SIII.1). However, seven other publications (nine patterns) did not provide adequate data to support this hypothesis (three cohort: [48,51,53]; two cross-sectional: [7,22]; two case–control: [8,64]). Furthermore, in one cohort study, contradictory findings emerged: although two dietary patterns (“Beans–vegetables” and “Rice–wheat–fruits”) characterized by a higher plant foods intake were identified, only the “Rice–wheat–fruits” pattern was related with a reduced GDM risk (Q3 vs. Q1, OR: 0.54, 0.36–0.83, *p*-trend = 0.010) [49].

✓ Miscellaneous Dietary Patterns

**Figure 10:**
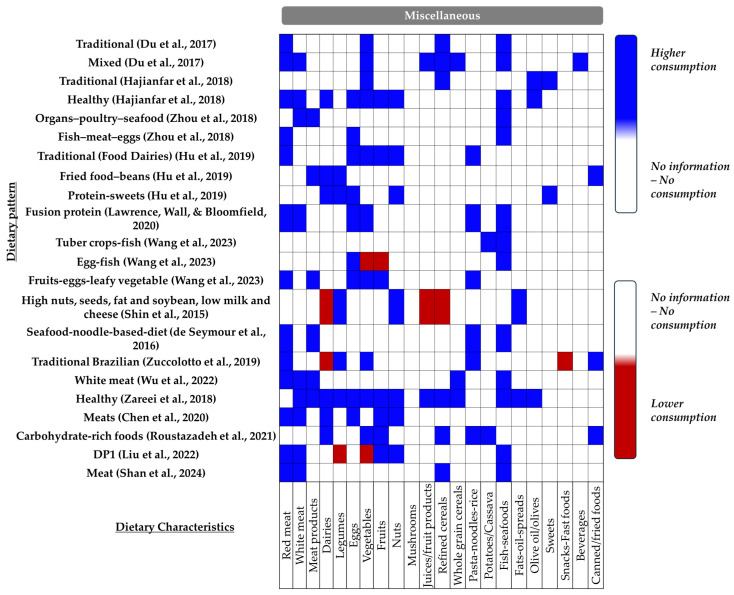
Combination of key food groups defining the Miscellaneous dietary patterns. Blue indicates higher consumption, while red indicates lower consumption [7,8,23,24,25,26,49,50,53,54,57,59,60,62,64].

Fifteen studies identified one or more Miscellaneous dietary patterns across the examined populations. These patterns are typically characterized by diverse combinations of food groups that do not fit neatly into other predefined categories, often reflecting specific cultural or regional dietary habits (Figure 10). 

As detailed in Table SIII.1, two of these studies—a cross-sectional survey by De Seymour et al. (2016) [54] and a case–control study by Zareei et al. (2018) [59]—found a protective association with the risk of GDM. In a similar vein, a prospective cohort study by Hu et al. (2018) involving 753 Chinese women noted that greater adherence to the “traditional” dietary pattern was associated with a lower risk of GDM (OR: 0.40, 95% CI: 0.23–0.71, *p* = 0.005) [50]. However, in the same study, other Miscellaneous patterns, including “fried food-beans” and “protein-sweets”, were not significantly associated with GDM risk [50]. Eight other studies reported no significant association between “miscellaneous” dietary patterns (*n* = 11) and GDM risk (three cohort: [24,26,53]; one cross-sectional: [7]; four case–control: [8,60,62,64]).

In contrast, Du et al. (2017), through a prospective cohort study, found that a higher adherence to the “traditional” dietary pattern—characterized by the elevated consumption of light-coloured vegetables, fine grain, red meat, tubers, and algae—was linked to an increased risk of GDM, with participants in the third and fourth quartiles showing significantly higher odds (Q3 vs. Q1: OR: 2.86, 95% CI: 1.19–6.83, *p* = 0.005; Q4 vs. Q1: OR: 2.92, 95% CI: 1.19–7.17, *p* = 0.005). However, this study did not find any significant associations between the “mixed” dietary pattern and GDM risk [23]. Similarly, Zhou et al. (2018) observed that only the “Fish-meat-eggs” dietary pattern had a positive relationship with GDM risk [49]. Additionally, two other cross-sectional studies [25,57] highlighted the potential negative impact of “Miscellaneous” dietary patterns on GDM risk.

### 3.5. Analysis of Stratified Data

**Figure 11:**
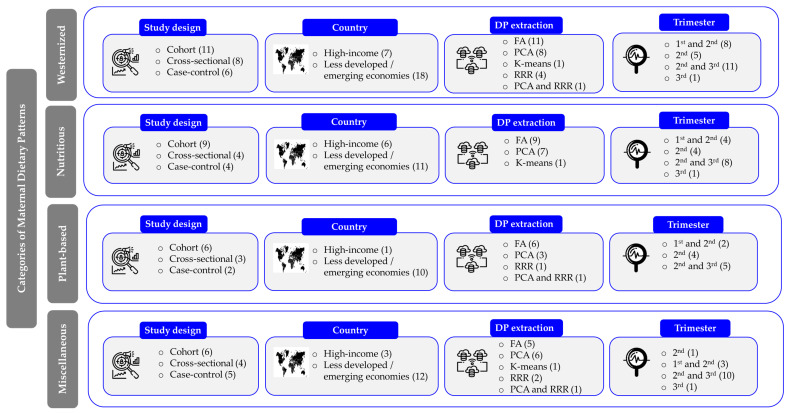
Stratification of studies by the study design and country, dietary pattern extraction methods, and trimester during which dietary assessments were carried out. The numbers in parenthesis indicate how many of the studies reported/presented that specific characteristic. FA: factor analysis; PCA: Principal Component Analysis; RRR: Reduced Rank Regression.

Figure 11 presents the stratification of the reviewed studies within each dietary pattern category (“Westernized”, “Nutritious”, “Plant-based”, and “Miscellaneous”, respectively) according to study design, country, dietary pattern extraction methodology, and the trimester of maternal dietary assessment. The studies reviewed were not categorized based on the dietary assessment tools employed, given that the FFQ was the most commonly used method for assessing maternal diet (utilized in 20 out of 28 studies).

The stratified analysis revealed variability in study design distribution both within and between dietary pattern categories. Cohort studies were the most commonly employed research approach, particularly prevalent within the “Nutritious” dietary pattern category. In this dietary pattern category, cohort studies were twice as common as cross-sectional and case–control studies. In contrast, the “Miscellaneous” category displayed a more balanced distribution—6 were cohort, 4 were cross-sectional and 5 were case–control—highlighting methodological diversity within this group.

The stratification of studies by countries with different income levels showed a significant focus on less developed countries/emerging economies across all dietary pattern categories. In the “Plant-based” pattern, almost all studies (*n* = 10) were conducted in these regions, with only one study from a Western setting [22]. Conversely, the “Nutritious” category, although still dominated by studies from low- and middle-income areas, exhibited a more balanced distribution, with six studies from these regions and eleven from higher-income settings.

Methodological discrepancies were noted both across and within dietary pattern categories, especially regarding the extraction methods and timing of dietary assessment during pregnancy, complicating cross-study comparisons. The figures in Supplementary Materials SIII (Figures SIII.1 and SIII.2) summarize the associations between dietary patterns and GDM risk, stratified by pattern categories, study design, country of origin, and assessment trimester. 

### 3.6. Region-Specific Analysis: The Case of China

**Figure 12:**
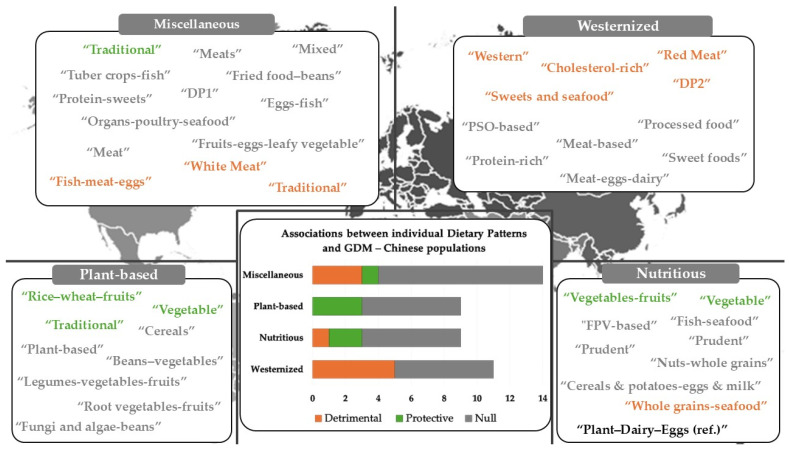
Overview of dietary patterns identified in the studies carried out in China. Dietary patterns marked in orange presented a detrimental link with GDM, those in green were protective against GDM, and those in grey showed no significant association with GDM risk.

We performed a focused analysis on studies conducted among Chinese populations, as most of the evaluated studies were conducted in China [8,23,45,48,49,50,52,53,57,62,63,64]. This approach aimed to provide a more comprehensive understanding of potential region-specific effects on the reported associations (Figure 12). 

Among the eleven “Westernized” dietary patterns, four were associated with an increased risk of GDM [23,57,62,63], while five showed no statistically significant associations [48,50,52,53,64]. Notably, in a cohort study, He et al. [45] found conflicting results (Table SIII.1). Furthermore, unlike findings from Australia [58], New Zealand [26], and Malaysia [51], none of the Chinese studies identified any protective effects for “Westernized” dietary patterns. 

As far as “Nutritious” dietary patterns are concerned, in Chinese populations, 67% of the patterns (six out of nine, (The “Plant–dairy–eggs” DP, in the cross-sectional study conducted by Wu et al. (2022) [57], was only used as a reference, and thus, it was not taken into consideration in the current analysis)) showed no relationship with GDM [23,45,49,50,52,64]. Only one study reported a detrimental effect (“Whole grain-seafood”) [50], while another two recorded a protective effect [8,64] (Figure 12). These findings align with global trends (Figure 6). 

Given that “plant-based” dietary patterns were mainly identified in studies conducted in China (nine out of fourteen), this focused analysis did not reveal any regional-specific trends. Similar observations were made in the “Miscellaneous” category.

## 4. Discussion

### 4.1. General Commentary

Based on the latest data from observational studies, this systematic review meticulously examines the intricate associations between maternal “a-posteriori” dietary patterns during pregnancy and the risk of GDM. Across 28 studies, over 90 dietary patterns were identified and categorized into four groups: “Westernized” (*n* = 35), “Nutritious” (*n* = 20), “Plant-based” (*n* = 14), and “Miscellaneous” (*n* = 22). Our findings do not establish definitive associations between maternal “a-posteriori” dietary patterns during pregnancy and GDM risk. Interestingly, “Westernized” dietary patterns, commonly identified in the reviewed studies, tended to be linked with an increased GDM risk. However, a very small portion of patterns within this category showed protective associations [26,51,58]. Conversely, “Plant-based” dietary patterns lacked detrimental associations and exhibited a tendency towards protective effects, with several patterns linked to a reduced risk of GDM. A similar trend was also reported in “Nutritious”, although a detrimental action was unexpectedly reported in one study [50]. However, as the authors suggested, this finding may not be directly attributed to the diet per se but rather to the high levels of toxic heavy metals and other environmental contaminants that have been proposed as additional risk factors of GDM and are often present in cereals and fish products that characterized this dietary pattern [50]. The “Miscellaneous” category showed a nearly equal distribution of patterns with detrimental [23,25,49,57] and protective [50,54,59] associations, pinpointing the absence of a clear directional trend regarding GDM risk. Notably, studies in Chinese populations largely align with the overall findings, mainly exhibiting a lack of solid associations with GDM risk. 

To date, numerous research studies, systematic reviews, and meta-analyses have indicated that “nutritious” dietary patterns reduce the likelihood of developing DM, whereas “unbalanced” dietary patterns increase the respective risk [65,66]. However, though this metabolic disorder shares several pathophysiological similarities with GDM [14], the results of existing systematic reviews and meta-analyses specific to GDM do not consistently demonstrate similar associations [27,28,29]. As already been emphasized, Kibret et al. (2018) [27], in a pooled estimate of six a-priori and a-posteriori studies [22,45,47,49,54,67], reported that a higher adherence to “healthy” dietary patterns reduced the risk of developing GDM (OR = 0.78; 95% CI: 0.56–0.99), whereas no links between “Western” dietary patterns and the odds of GDM were described (OR = 0.94; 95% CI: 0.81–1.07). A 2020 systematic review and meta-analysis analyzing 13 cohort studies involving over 92,000 women suggested that “Prudent” dietary patterns decreased the GDM risk (RR: 0.78, 95%CI: 0.63–0.96), while “Western” dietary patterns heightened it (RR: 1.27, 95 CI %: 1.03–1.56). However, these associations did not persist when the analysis was stratified according to the time of diet assessment (before or during pregnancy) [28]. More recently, Haghighatdoost et al. (2022) revealed that only “unhealthy” dietary patterns during pregnancy were significantly associated with GDM (RR: 1.21, 95% Cl: 0.83–1.76) [29]. 

The critical evaluation of our findings against the existing literature underscores that we are still far from elucidating the exact role of maternal “a-posteriori” dietary patterns during pregnancy in GDM incidence and highlights the necessity of further studies in this field. In the next section, we will explore the potential factors contributing to the lack of clear associations between expectant mothers’ dietary habits and GDM risk. Additionally, we will provide insights into potential areas for future research.

### 4.2. Exploring the Underlying Causes for the Observed Lack of Associations Between Maternal A-Posteriori Dietary Patterns and GDM

GDM is influenced by a complex interplay of genetic, epigenetic, and environmental factors [3,68]. A family history of DM is a significant independent risk factor [69,70], while there is evidence of common risk gene polymorphisms for DM and GDM [70,71]. In this context, numerous research efforts have explored the association between gene polymorphisms and the risk of developing GDM across different regions worldwide. These studies indicate that while some genes are widely prevalent, others are more common within specific ethnicities or geographic areas [3,72,73]. This could partially explain why certain populations, such as Asian and Hispanic women, exhibit a higher prevalence of GDM compared to other ethnic groups [2,71]. However, genetic variants account for only a portion of the GDM risk, and their precise role remains unclear [3,74]. Additionally, various individual characteristics also appear to contribute to the manifestation of GDM [75]. Excessive GWG and maternal obesity enhance insulin resistance and increase the risk of GDM, potentially due to their effect on the pro-inflammatory state and placental dysfunction [13,76]. Furthermore, certain physiological traits linked to historical nutrient-poor intrauterine environments and fetal programming, such as the higher body fat percentage observed among Asian women [58], are also associated with the manifestation of GDM [76,77,78]. Thus, isolating the impact of diet alone remains a significant challenge.

In addition, the unique food cultures, socio-economic conditions, and lifestyle characteristics of the studied populations may also contribute to the inconsistency observed across studies [4,68,79]. In less developed nations, traditional diets often rely on a high-glycaemic index and nutrient-poor staples such as rice and noodles [24,50,54,80]. However, depending on the socio-economic status and regional characteristics [48,64,80,81], these foods may be consumed alongside nutrient-rich and potential anti-diabetic ingredients such as deep-sea fish, legumes, and seaweeds, significantly differentiating the nutritional profile [50,82,83]. Additionally, the “Westernized” diet is not uniform globally, encompassing variations influenced by food availability, the retention of local dietary characteristics, and varying levels of food processing, complicating the ability to draw firm conclusions [79,81]. 

The different statistical techniques used to identify dietary patterns could also contribute to the absence of significant findings [19]. The RRR method is considered effective for linking dietary patterns to specific health outcomes, while PCA offers a broader dietary structure analysis [53,62]. Thus, further research is needed to clarify the impact of different methods on outcomes. However, even when using the same methodologies, studies often show significant heterogeneity or lack sufficient details regarding data pre-treatment, the number of food groups analyzed, specific food items included in food groups, and portion sizes consumed. Additionally, most of the publications reviewed did not provide data on the study populations’ nutritional status and dietary quality (see Supplementary Materials SIV). In this frame, Wu et al. (2022) provided valuable insights into the exact role of different dietary patterns in pregnancy complications, demonstrating that different protein sources have varying impacts on GDM risk [57]. 

### 4.3. Potential Strengths and Limitations

Our systematic review exhibits several strengths and possible limitations. One key aspect of our systematic review is that we exclusively focus on “a-posteriori-derived” dietary patterns. It is generally accepted that, compared to traditional approaches, “a-posteriori-derived” analysis uniquely captures real-world, culturally specific dietary behaviours and provides a more holistic and personalized appraisal of the potential association between maternal diet and GDM manifestation [20,30]. Furthermore, to comprehensively evaluate the existing research, we extensively searched multiple databases (PubMed, Science Direct, Web of Science, Google Scholar, Scopus) using the PRISMA 2020 criteria [31]. This literature review synthesizes the most updated data from 28 observational studies, covering an extensive sample size of pregnant women (*n* = 39,735) across 12 countries, providing a broad perspective on maternal dietary patterns during pregnancy and GDM risk. Notably, from the twenty-eight original articles evaluated, eleven were published after March 2020, which was the cut-off period of Haghighatdoost et al. (2022) [29], and were not previously assessed within the settings of a systematic review [8,52,53,56,57,58,60,61,62,63,64]. A notable strength of our review is the stratified analysis conducted within each dietary pattern category, which evaluates potential sources of heterogeneity, such as study design, geographic location, dietary pattern extraction methodology, and timing of dietary assessment. This approach allows for a more nuanced understanding of the complex associations between maternal dietary patterns and GDM risk, setting our review apart from previous studies. Meanwhile, according to the NOS, most studies evaluated were rated high quality, further supporting the reliability of our findings.

Although the research included in our analysis was observational, making the included studies prone to bias and confounding factors [84], it is noteworthy that half of the studies were cohort studies. Cohort studies are widely regarded as the most effective observational approach for exploring potential diet–health associations because they are less susceptible to recall or selection biases [1,43]. A meta-analysis was also not feasible due to substantial variations in research design, population characteristics, methodology, and dietary assessment periods among the surveys. 

## 5. Conclusions

The study concluded that the relationship between maternal “a posteriori” dietary patterns during pregnancy and the risk of GDM is complex and influenced by multiple factors, including genetic, environmental, and sociocultural contexts. Although most of the studies did not reveal statistically significant associations between maternal dietary patterns during pregnancy and GDM, “Westernized” dietary patterns tended to be associated with an increased risk of GDM. Conversely, both “Nutritious” and “Plant-based” dietary patterns generally lacked detrimental associations and appeared beneficial in specific populations, indicating potential areas for preventive dietary recommendations. The “Miscellaneous” category displayed varied effects, with some studies highlighting beneficial impacts while others indicated a potentially harmful role, underscoring the absence of a consistent trend in GDM risk. Given the mixed findings, there is an apparent necessity for further research to explore the potential influence of various genetic and sociocultural backgrounds on GDM risk and to standardize research methodologies, with the ultimate goal of developing more effective dietary guidelines and interventions for preventing GDM. Furthermore, a more detailed assessment of nutritional status in these studies could facilitate the development of tailored nutritional recommendations to mitigate GDM risk, thus contributing to better maternal and fetal health outcomes.

## Figures and Tables

**Table 1:** Search strategy terms applied in each electronic database for conducting the present systematic review.

Electronic Database	Terms Syntax	Filters
PubMed	(“Gestational diabetes mellitus”) AND (“Dietary pattern”) [all fields]; (“Gestational diabetes mellitus”) AND (“Dietary habit”) [all fields]; (“Gestational diabetes mellitus”) AND (“Maternal Diet”) [all fields]; (“Gestational diabetes mellitus”) AND (“a-posteriori”) [all fields]; (“Gestational diabetes mellitus”) AND (“Eating habit”) [all fields]; ((dietary patterns) AND (pregnancy)) AND (GDM) [all fields]	Year: - Species: Humans Age: Adult: 19+ years
Science Direct	(“Gestational diabetes mellitus”) AND (“Dietary pattern”) [all fields]; (“Gestational diabetes mellitus”) AND (“Dietary habit”) [all fields]; (“Gestational diabetes mellitus”) AND (“Maternal Diet”) [all fields]	Year: - Article type: Review Research articles
Web of Science	(“Gestational diabetes mellitus”) AND (“Dietary pattern”) [all fields]; (“Gestational diabetes mellitus”) AND (“Dietary habit”) [all fields]; (“Gestational diabetes mellitus”) AND (“Maternal Diet”) [all fields]	Year: - Article type: Review Research articles
Scopus	(“Gestational diabetes mellitus”) AND (“Dietary pattern”) [Article/abstract/keywords]; (“Gestational diabetes mellitus”) AND (“Dietary habit”) [Article/abstract/keywords]; (“Gestational diabetes mellitus”) AND (“Maternal Diet”) [Article/abstract/keywords]	Year: - Article type: Review Research articles

**Table 2:** Key characteristics of the studies included in the present systematic review (*n* = 28).

Study	Country	Sample Size (*n*)	Follow-Up (Week)	Dietary Assessment Tool	Dietary Patterns Extraction Method	GDM Diagnosis			Confounding Factors
						Diagnostic Tool	Criteria	Prevalence (%)	
Cohort Studies									
Radesky et al. (2007) The “Project Viva” [44]	USA	1733	N/A	(v) FFQ	FA	50 g, 1 h OGCT ^^^	ACOG	5	Maternal age, pp-BMI, race, family history of DM, previous history of GDM smoking.
He et al. (2015) [45]	China	3063	8–14 w	(v) FFQ	FA	75 g, 2 h OGTT	IADPSG	18	Maternal age, education, monthly income, parity, pp-BMI, family history of DM
Tryggvadottir et al. (2015) [46]	Iceland	168	4–9 w	4 d FR	PCA	75 g, 2 h OGTT	WHO	10	Maternal age, parity, pp-weight, EI, weekly GWG, total MET
Nascimento et al. (2016) [47]	Brazil	838	Until delivery	(v) FFQ	FA	75 g, 2 h OGTT	IADPSG	11	Maternal age, education, pp-BMI, family history of DM, parity
Du et al. (2017) [23]	China	753	9–23 w	24 hR (x2)	PCA	Medical records	IADPSG	9	Maternal age, pp-BMI, education, partner smoking, family history of DM, parity, daily EI, PA
Hajianfar et al. (2018) [24]	Iran	812	8–20 w	(v) FFQ	FA	50 g, 1 h OGCT	N/A	N/A	Maternal age, EI, BMI, SES, PA
Mak et al. (2018) [48]	China	1337	4–13 w	(v) FFQ	FA	75 g, 2 h OGTT	IADPSG	15	Maternal age, pp-BMI, family history of DM, parity, education, PA
Zhou et al. (2018) [49] The “Tongji Maternal and Child Health Cohort”	China	2755	2 w	(v) FFQ	PCA	75 g, 2 h OGTT	IADPSG	9	Maternal age, ethnicity, education, average personal income, family history of DM, family history of obesity, smoking, alcohol, parity, pp-BMI, GWG before GDM diagnosis, other dietary patterns, EI
Hu et al. (2019) The “Born in Shenyang Cohort Study” [50]	China	1014	~2 w	3 d FD and FFQ	FA	75 g, 2 h OGTT	IADPSG	24	Maternal age, pp-BMI, parity, family income, education, ethnicity, smoking, EI, PA, other dietary patterns
Lawrence, Wall, & Bloomfield (2020) The “Growing Up in New Zealand Study” [26]	New Zealand	5384	N/A	FFQ	PCA	Coded clinical data	NZSSD	5	Maternal age, ethnicity, NZDep06 score, pp-BMI, pp and 1st trimester PA, smoking, alcohol, alternative DP, maternal socioeconomic deprivation
Yong et al. (2020) The “Malaysian SECOST Cohort” [51]	Malaysia	452	~14 w	(v) FFQ	PCA	75 g, 2 h OGTT	Ministry of Health Malaysia guidelines	11	Maternal age, ethnicity, history of GDM and family history of DM
De Seymour et al. (2022) [52] The “Complex Lipids in Mothers and Babies Cohort”	China	1437	Every trimester until 1 year postpartum	FFQ	FA	75 g, 2 h OGTT	IADPSG	28	Maternal age, offspring gender, EI, BMI, CLIMB treatment group, education, family income, ethnicity
Wang et al. (2023) The “Tianjin Birth Cohort” [53]	China	2202	N/A	(v) FFQ	PCA and RRR	75 g, 2 h OGTT	IADPSG	17	Maternal age, pp-BMI, ethnicity, education, monthly incomes, parity, family history of DM, smoking, alcohol, PA, EI
Cross-Sectional Studies									
Shin et al. (2015) The “NHANES Study” [25]	USA	249	-	24 hR	RRR	FBG, insulin, HOMA-IR	IADPSG	14	Maternal age, pp-BMI, GWG, ethnicity, family poverty, income, education, marital status, CRP, PA
De Seymour et al. (2016) “The GUSTO Study” [54]	Singapore	909	-	24 hR	FA	OGTT	WHO	18	Maternal age, BMI, EI, household income, previous history of GDM, family history of DM, ethnicity, education, birth order, smoking, alcohol intake
Flynn et al. (2016) Part of the “UPBEAT Randomized Controlled Trial” [22]	UK	1023	-	FFQ	FA	75 g, OGTT	IADPSG	23	Maternal age, parity, ethnicity, BMI, living in a deprived area, treatment allocation
Sartorelli et al. (2019) [55]	Brazil	785	-	24 hR (x2) ◊	RRR	75 g, 2 h OGTT	WHO	18	Maternal age, education, smoking, PA, parity, history of GDM and family history of DM, GWG-GA, EI, dietary underreporting
Zuccolotto et al. (2019) [7]	Brazil	785	-	24 hR (x2)	PCA	75 g, 2 h OGTT	WHO	18	Maternal age, GA, previous GDM, education, family history of DM, smoking, PA, number of children, excessive body weight
Pajunen et al. (2022) [56]	Finland	351	-	3 d FD	PCA	75 g, 2 h OGTT	Finnish Current Care Guidelines	23	Pp-BMI, original trial intervention group *
Wu et al. (2022) [57]	China	1014	-	(v) FFQ	K-means CA	75 g, 2 h OGTT	IADPSG	19	Maternal age, education, monthly household income, GA, pp-BMI, family history of DM, history of GDM, smoking, alcohol intake, PA, EI, fibre, PI:EI, CI:EI, FI:EI, cholesterol
Ebrahimi et al. (2024) The “Creatine and Pregnancy Outcomes Study” [58]	Australia	215	-	(v) FFQ	PCA	75 g, 2 h OGTT	Australian Diabetes in Pregnancy Society guidelines	10	Maternal age, ethnicity, BMI
Case–Control Studies									
Zareei et al. (2018) [59]	Iran	104 GDM and 100 controls	-	(v) FFQ	PCA	50 g, 1 h OGCT	N/A	-	Maternal age, education, BMI, GWG, number of deliveries, GDM history, job, PA
Chen et al. (2020) [8]	China	1464 GDM and 8092 controls	-	(v) FFQ	FA	Medical records	IADPSG	-	Maternal age, education, BMI, GA, alcohol intake, smoking, parity, gestational hypertension, preterm, GWG, family history of GDM, EI
Roustazadeh et al. (2021) [60]	Iran	306 GDM and 387 controls	-	(v) FFQ	PCA	75 g, 2 h OGTT	ADA	-	Maternal age, pp-BMI, GWG, energy intake, SES, education, PA, number of pregnancies
Waheby et al. (2021) [61]	Gaza strip	70 GDM and 140 controls	-	(v) FFQ	FA	75 g, 2 h OGTT	WHO	-	Family history of DM, CVD, hyperlipidaemia, PA, GWG, FBG
Liu et al., (2022) [62]	China	143 GDM and 345 controls	-	FFQ	RRR	75 g, 2 h OGTT	China’s Guidelinesfor Diagnosis and Treatment of GDM	-	Maternal age, education, number of pregnancies, pp-BMI, GWG, EI, and supplement intake (folic acid, B_12_, and B_6_)
Cui et al. (2023) [63]	China	372 GDM and 744 controls	-	(v) FFQ	RRR	75 g, 2 h OGTT	IADPSG	-	Maternal age, pp-BMI, ethnicity, primiparity, per capita monthly income, education, family history of DM and obesity, smoking, drinking, PA, insomnia, GWG, EI, SFA, MUFA, PUFA
Shah et al. (2024) [64]	China	107 GDM and 78 controls	-	FFQ	FA	75 g, 2 h OGTT	IADPSG	-	Maternal age, gestational week, pp-BMI, parity, passive smoking, other dietary patterns and EI.

^: For participants (*n* = 39) with incomplete glucose testing data, the identification of GDM cases was based on clinical medical records. ◊ The 2nd recall was obtained only for the 73% of the sample. * This study was part of an intervention, with fish oil, probiotics, both, or placebo, that did not affect the onset of GDM. (v): Validated; 24 hR: 24-Hour Recalls; ADA: American Diabetes Association; ACOG: American College of Obstetricians and Gynecologists; BMI: Body Mass Index; CA: Cluster Analysis; CI: Carbohydrates Intake; CLIMB: Complex Lipids In Mothers and Babies Cohort; CRP: C-reactive protein; CVD: Cardiovascular Diseases; d: Day; DM: Diabetes Mellitus; DP: Dietary Pattern; EI: Energy Intake; FA: Factor Analysis; FBG: Fasting Blood Glycose; FD: Food Dairies; FFQ: Food Frequency Questionnaire; FI: Fat Intake; FR: Food Records; GA: Gestational Age; GDM: Gestational Diabetes Mellitus; GWG: Gestational Weight Gain; h: hours; HOMA-IR: Homeostatic Model Assessment for Insulin Resistance; IADPSG: International Association of Diabetes and Pregnancy; MET: Metabolic Equivalent of Task; MUFA: Monounsaturated Fatty Acids; N/A: Not Available; Nzdep06: New Zealand Deprivation Index; NZSSD: New Zealand Society for the Study of Diabetes; OGCT: Oral Glucose Challenge Test; OGTT: Oral Glucose Tolerance Test; PA: Physical Activity; PCA: Principal Component Analysis; PI: Protein Intake; pp: Pre-Pregnancy; PUFA: Polyunsaturated Fatty Acids; RRR: Reduced Rank Regression, SFA: Saturated Fatty Acids; SES: Socio-Economical Status; w: Week; WHO: World Health Organization.

## Data Availability

All data are available within the article and Supplementary Materials.

## Supplementary Materials

The following supporting information can be downloaded at https://www.mdpi.com/article/10.3390/nu16213632/s1, Table SI.1. Newcastle—Ottawa Quality Assessment Scale for Cohort Studies; Table SI.2. Newcastle—Ottawa Quality Assessment Scale for Cross-sectional Studies; Table SI.3. Newcastle—Ottawa Quality Assessment Scale for Case–control studies; Table SII.1. Quality Assessment based on the Newcastle–Ottawa Scale of the 13 Cohort Studies under review; Table SII.2. Quality Assessment based on the Newcastle–Ottawa Scale of the 8 Cross-sectional Studies under review; Table SII.3. Quality Assessment based on the Newcastle–Ottawa Scale of the 7 case–control Studies under review; Table SIII.1. Overview of the dietary patterns and main findings on the links between maternal dietary patterns and gestational diabetes mellitus (*n* = 28). Only the most adjusted models are presented. To facilitate data interpretation, statistically significant data are presented in bold letters. The orange colour was used to depict the detrimental effect, the green to illustrate the protective effect, and the light grey to represent the null effect of a specific DP; Figure SIII.1 Associations between dietary patterns (DP) and risk of gestational diabetes mellitus. The findings within each DP category, expressed as a percentage of DP, are stratified by the study design and country, dietary pattern extraction methods, and trimester during which dietary assessments were carried out. FA: factor analysis; PCA: Principal Component Analysis; RRR: Reduced Rank Regression; Figure SIII.2 Associations between maternal dietary patterns, during pregnancy, and risk of gestational diabetes mellitus. The findings within each dietary pattern category are stratified by the study design and country, dietary pattern extraction methods, and trimester during which dietary assessments were carried out. Withing the parenthesis are given the number of dietary patterns with Detrimental (D); Null (N), and Protective (P) associations. FA: factor analysis; PCA: Principal Component Analysis; RRR: Reduced Rank Regression; Table SIV.1. Key methodological parameters of the studies under evaluation (*n* = 28).
