# Supplementary material for: Are Maternal Dietary Patterns During Pregnancy Associated with the Risk of Gestational Diabetes Mellitus? A Systematic Review of Observational Studies
Source: Nutrients. 2024 Oct 25;16(21):3632. doi: 10.3390/nu16213632 (PMC11547687; doi:10.3390/nu16213632)
Supplement: Supplementary file 1 [file nutrients-16-03632-s001.zip › nutrients-3274816 suppl materials SII.pdf]

Supplementary Material II

Table SII.1. Quality Assessment based on the Newcastle-Ottawa Scale of the 13 Prospective Cohort Studies under review.

References	1. Selection				2. Comparability	3. Outcome			Sum
	I. Representativeness of the Sample	II. Selection of non-exposed	III. Ascertainment of exposure	IV. Outcome of interest	V. Comparability	VI. Assessment of the Outcome	VII. Duration of Follow-up	VIII. Rate of Follow-up	
**Radesky et al. [2008]** [1]	1	1	1	1	2	1	1	1	**9**
**He et al. [2015]** [2]	1	1	1	1	2	1	1	1	**9**
**Tryggvadottir et al. [2015]** [3]	1	1	0	1	2	1	1	1	**8**
**Nascimento et al. [2016]** [4]	1	1	1	1	2	1	1	1	**9**
**Du et al. [2017]** [5]	1	1	0	1	2	1	1	1	**8**
**Hajianfar et al. [2018]** [6]	1	1	1	1	1	1	1	0	**7**
**Mak et al. [2018]** [7]	1	1	1	1	2	1	1	0	**8**
**Zhou et al. [2018]** [8]	1	1	1	1	2	1	1	1	**9**
**Hu et al. [2019]** [9]	1	1	1	1	2	1	1	1	**9**
**Lawrence, Wall, and Bloomfield, [2020]** [10]	1	1	0	0	1	1	1	1	**6**
**Yong et al. [2020]** [11]	1	1	1	1	2	1	1	1	**9**
**De Seymour et al. [2022]** [12]	1	1	0	0	1	1	1	0	**5**
**Wang et al. [2023]** [13]	1	1	1	1	2	1	0	0	**7**
**Adequate (%)**	100	100	69	85	77	100	92	69	
**Inadequate (%)**	0	0	31	15	0	0	8	31	
**Unclear (%)**	0	0	0	0	23	0	0	0	

Table SII.2. Quality Assessment based on the Newcastle-Ottawa Scale of the 8 Cross-sectional Studies under review.

References	1. Selection				2. Comparability	3. Outcome		Sum
	I. Representative-ness of the Sample	II. Sample size	III. Non-respondents	IV. Ascertain-ment of the exposure	V. Comparability	VI. Assessment of the Outcome	VII. Statisti-cal test	
**Shin et al. [2015]** [14]	1	0	1	1	1	2	1	**7**
**De Seymour et al. [2016]** [15]	1	0	1	1	2	2	1	**8**
**Flynn et al. [2016]** [16]	1	0	1	2	1	1	1	**6**
**Sartonelli et al. [2019]** [17]	1	1	0	1	2	2	1	**8**
**Zuccolotto et al. [2019]** [18]	1	1	0	1	2	2	1	**8**
**Pajunen et al. [2022]** [19]	0	0	1	1	0	2	1	**5**
**Wu et al. [2022]** [20]	1	1	0	2	2	2	1	**9**
**Ebrahimi et al. [2024]** [21]	1	0	0	2	1	2	1	**7**
**Adequate (%)**	88	37,5	50	37,5	50	87,5	100	
**Inadequate (%)**	13	62,5	50	0	12,5	0	0	
**Unclear (%)**	0	0	0	62,5	37,5	12,5	0	

Table SII.3. Quality Assessment based on the Newcastle-Ottawa Scale of the 7 case-control Studies under review.

References	1. Selection				2. Comparability	3. Exposure			Sum
	**I. Case adequate definition**	**II. Representative-ness of the Sample**	**III. Controls selection**	**IV. Defini-tion of con-trols**	**V. Comparability**	**VI. Ascertain-ment of expo-sure**	**VII. Method of As-certainment (cases vs. controls)**	**VIII. Non-re-sponse rate**	
**Zareei et al. [2018]** [22]	1	0	1	1	2	1	1	0	**7**
**Chen et al. [2020]** [23]	1	1	1	1	2	1	1	0	**8**
**Roustazadeh et al. [2021]** [24]	1	1	1	1	1	1	1	1	**8**
**Waheby et al. [2021]** [25]	1	1	1	1	2	1	1	1	**9**
**Liu et al. [2022]** [26]	1	0	1	1	1	0	1	0	**5**
**Cui et al. [2023]** [27]	1	1	1	1	2	1	1	0	**8**
**Shan et al. [2024]** [28]	1	0	1	1	1	0	1	0	**5**
**Adequate (%)**	100	57	100	100	57	71	100	29	
**Inadequate (%)**	0	43	0	0	0	29	0	71	
**Unclear (%)**	0	0	0	0	43	0	0	0	

References

1. Radesky, J.S.; Oken, E.; Rifas-Shiman, S.L.; Kleinman, K.P.; Rich-Edwards, J.W.; Gillman, M.W. Diet during Early Pregnancy and Development of Gestational Diabetes. *Paediatric and Perinatal Epidemiology* **2007**, *22*, 47–59, doi:https://doi.org/10.1111/j.1365-3016.2007.00899.x.
2. He, J.-R.; Yuan, M.-Y.; Chen, N.-N.; Lu, J.-H.; Hu, C.-Y.; Mai, W.-B.; Zhang, R.-F.; Pan, Y.-H.; Qiu, L.; Wu, Y.-F.; et al. Maternal Dietary Patterns and Gestational Diabetes Mellitus: A Large Prospective Cohort Study in China. *British Journal of Nutrition* **2015**, *113*, 1292–1300, doi:https://doi.org/10.1017/s0007114515000707.
3. Tryggvadottir, E.A.; Medek, H.; Birgisdottir, B.E.; Geirsson, R.T.; Gunnarsdottir, I. Association between Healthy Maternal Dietary Pattern and Risk for Gestational Diabetes Mellitus. *European Journal of Clinical Nutrition* **2015**, *70*, 237–242, doi:https://doi.org/10.1038/ejcn.2015.145.
4. Nascimento, G.R.; Alves, L.V.; Fonseca, C.L.; Figueiroa, J.N., Alves, J.G. Dietary Patterns and Gestational Diabetes Mellitus in a Low Income Pregnant Women Population in Brazil - a Cohort Study. *Archivos Latinoamericanos de Nutrition*, **2016,** 66(4), 1–8.
5. Du, H.Y.; Jiang, H.; O, K.; Chen, B.; Xu, L.J.; Liu, S.P.; Yi, J.P.; He, G.S.; Qian, X. Association of Dietary Pattern during Pregnancy and Gestational Diabetes Mellitus: A Prospective Cohort Study in Northern China. *Biomedical and environmental sciences: BES* **2017**, *30*, 887–897, doi:https://doi.org/10.3967/bes2017.119.
6. Hajianfar, H.; Esmaillzadeh, A.; Feizi, A.; Shahshahan, Z.; Azadbakht, L. The Association between Major Dietary Patterns and Pregnancy-Related Complications IRANIAN MEDICINE. *Arch Iran Med* **2018**, *21*, 443–451.
7. Mak, J.K.L.; Pham, N.M.; Lee, A.H.; Tang, L.; Pan, X.-F.; Binns, C.W.; Sun, X. Dietary Patterns during Pregnancy and Risk of Gestational Diabetes: A Prospective Cohort Study in Western China. *Nutrition Journal* **2018**, *17*, doi:https://doi.org/10.1186/s12937-018-0413-3.
8. Zhou, X.; Chen, R.; Zhong, C.; Wu, J.; Li, X.; Li, Q.; Cui, W.; Yi, N.; Xiao, M.; Yin, H.; et al. Maternal Dietary Pattern Characterised by High Protein and Low Carbohydrate Intake in Pregnancy Is Associated with a Higher Risk of Gestational Diabetes Mellitus in Chinese Women: A Prospective Cohort Study. *British Journal of Nutrition* **2018**, *120*, 1045–1055, doi:https://doi.org/10.1017/s0007114518002453.
9. Hu, J.; Oken, E.; Aris, I.; Lin, P.-I.; Ma, Y.; Ding, N.; Gao, M.; Wei, X.; Wen, D. Dietary Patterns during Pregnancy Are Associated with the Risk of Gestational Diabetes Mellitus: Evidence from a Chinese Prospective Birth Cohort Study. *Nutrients* **2019**, *11*, 405, doi:https://doi.org/10.3390/nu11020405.
10. Lawrence, R.L.; Wall, C.R.; Bloomfield, F.H. Dietary Patterns and Dietary Adaptations in Women with and without Gestational Diabetes: Evidence from the Growing up in New Zealand Study. *Nutrients* **2020**, *12*, 227, doi:https://doi.org/10.3390/nu12010227.
11. Yong, H.Y.; Mohd Shariff, Z.; Mohd Yusof, B.-N.; Rejali, Z.; Appannah, G.; Bindels, J.; Tee, Y.Y.S.; van der Beek, E.M. The Association between Dietary Patterns before and in Early Pregnancy and the Risk of Gestational Diabetes Mellitus (GDM): Data from the Malaysian SECOST Cohort. *PLOS ONE* **2020**, *15*, e0227246, doi:https://doi.org/10.1371/journal.pone.0227246.
12. de Seymour, J.V.; Beck, K.L.; Conlon, C.A.; Jones, M.B.; Colombo, J.; Xia, Y.-Y.; Han, T.-L.; Qi, H.-B.; Zhang, H.; Baker, P.N. An Investigation of the Relationship between Dietary Patterns in Early Pregnancy and Maternal/Infant Health Outcomes in a Chinese Cohort. *Frontiers in Nutrition* **2022**, *9*, doi:https://doi.org/10.3389/fnut.2022.775557.
13. Wang, S.; Liu, H.; Luo, C.; Zhao, R.; Zhou, L.; Huang, S.; Ge, Y.; Cui, N.; Shen, J.; Yang, X.; et al. Association of Maternal Dietary Patterns Derived by Multiple Approaches with Gestational Diabetes Mellitus: A Prospective Cohort Study. *International Journal of Food Sciences and Nutrition* **2023**, *74*, 487–500, doi:https://doi.org/10.1080/09637486.2023.2220082.
14. Shin, D.; Lee, K.; Song, W. Dietary Patterns during Pregnancy Are Associated with Risk of Gestational Diabetes Mellitus. *Nutrients* **2015**, *7*, 9369–9382, doi:https://doi.org/10.3390/nu7115472.
15. de Seymour, J.; Chia, A.; Colega, M.; Jones, B.; McKenzie, E.; Shirong, C.; Godfrey, K.; Kwek, K.; Saw, S.-M.; Conlon, C.; et al. Maternal Dietary Patterns and Gestational Diabetes Mellitus in a Multi-Ethnic Asian Cohort: The GUSTO Study. *Nutrients* **2016**, *8*, 574, doi:https://doi.org/10.3390/nu8090574.
16. Flynn, A.C.; Seed, P.T.; Patel, N.; Barr, S.; Bell, R.; Briley, A.L.; Godfrey, K.M.; Nelson, S.M.; Oteng-Ntim, E.; Robinson, S.M.; et al. Dietary Patterns in Obese Pregnant Women; Influence of a Behavioral Intervention of Diet and Physical Activity in the UPBEAT Randomized Controlled Trial. *International Journal of Behavioral Nutrition and Physical Activity* **2016**, *13*, doi:https://doi.org/10.1186/s12966-016-0450-2.
17. Sartorelli, D.S.; Zuccolotto, D.C.C.; Crivellenti, L.C.; Franco, L.J. Dietary Patterns during Pregnancy Derived by Reduced-Rank Regression and Their Association with Gestational Diabetes Mellitus. *Nutrition* **2019**, *60*, 191–196, doi:https://doi.org/10.1016/j.nut.2018.10.008.
18. Zuccolotto, D.C.C.; Crivellenti, L.C.; Franco, L.J.; Sarotelli, D.S. Dietary Patterns of Pregnant Women, Maternal Excessive Body Weight and Gestational Diabetes. *Revista de Saúde Pública* **2019**, *53:52*, 52, doi:https://doi.org/10.11606/s1518-8787.2019053000909.
19. Pajunen, L.; Korkalo, L.; Koivuniemi, E.; Houttu, N.; Pellonperä, O.; Mokkala, K.; Shivappa, N.; Hébert, J.; Vahlberg, T.; Tertti, K.; et al. A Healthy Dietary Pattern with a Low Inflammatory Potential Reduces the Risk of Gestational Diabetes Mellitus Keywords Gestational Diabetes Mellitus · Dietary Pattern · Dietary Inflammatory Index · Saturated Fatty Acid Abbreviations BMI Body Mass Index DII Dietary Inflammatory Index E-DII Energy-Adjusted Dietary Inflammatory Index

GDM Gestational Diabetes Mellitus IDQ Index of Diet Quality MET Metabolic Equivalent Index for Physical Activity OGTT Oral Glucose Tolerance Test SFA Saturated Fatty Acid. *Trial registration ClinicalTrials.gov Identifier: NCT01922791* **2022**, *61*, 1477–1490, doi:https://doi.org/10.1007/s00394-021-02749-z.
20. Wu, W.; Tang, N.; Zeng, J.; Jing, J.; Cai, L. Dietary Protein Patterns during Pregnancy Are Associated with Risk of Gestational Diabetes Mellitus in Chinese Pregnant Women. *Nutrients* **2022**, *14*, 1623, doi:https://doi.org/10.3390/nu14081623.
21. Ebrahimi, S.; Ellery, S.J.; Leech, R.M.; van der Pligt, P.F. Associations between Diet Quality and Dietary Patterns and Gestational Diabetes Mellitus in a Low-Risk Cohort of Pregnant Women in Australia: A Cross-Sectional Study. *Journal of Human Nutrition and Dietetics* **2024**, *37*, 503–513, doi:https://doi.org/10.1111/jhn.13274.
22. Zareei, S.; Homayounfar, R.; Naghizadeh, M. mehdi; Ehrampoush, E.; Rahimi, M. Dietary Pattern in Pregnancy and Risk of Gestational Diabetes Mellitus (GDM). *Diabetes & Metabolic Syndrome: Clinical Research & Reviews* **2018**, *12*, 399–404, doi:https://doi.org/10.1016/j.dsx.2018.03.004.
23. Chen, Q.; Wu, W.; Yang, H.; Zhang, P.; Feng, Y.; Wang, K.; Wang, Y.; Wang, S.; Zhang, Y. A Vegetable Dietary Pattern Is Associated with Lowered Risk of Gestational Diabetes Mellitus in Chinese Women. *Diabetes & Metabolism Journal* **2020**, *44*, 887–896, doi:https://doi.org/10.4093/dmj.2019.0138.
24. Roustazadeh, A.; Mir, H.; Jafarirad, S.; Mogharab, F.; Hosseini, S.A.; Abdoli, A.; Erfanian, S. A Dietary Pattern Rich in Fruits and Dairy Products Is Inversely Associated to Gestational Diabetes: A Case-Control Study in Iran. *BMC Endocrine Disorders* **2021**, *21*, doi:https://doi.org/10.1186/s12902-021-00707-8.
25. Wahedy, kanan; El Bilbeisi, A.H.; Bakry, M. Dietary Patterns and Their Association with Glycemic Control and Risk of Gestational Diabetes Mellitus in Gaza Strip, Palestine: A Case Control Study. *Bulletin of Pharmaceutical Sciences. Assiut University* **2021**, *44*, 537–549, doi:https://doi.org/10.21608/bfsa.2021.90526.1147.
26. Liu, Y.; Lu, L.; Yi, M.; Shen, C.; Lu, G.; Jia, J.; Wu, H. Study on the Correlation between Homocysteine-Related Dietary Patterns and Gestational Diabetes Mellitus:a Reduced-Rank Regression Analysis Study. *BMC Pregnancy and Childbirth* **2022**, *22*, doi:https://doi.org/10.1186/s12884-022-04656-5.
27. Cui, N.; Li, Y.; Huang, S.; Ge, Y.; Guo, S.; Tan, L.; Hao, L.; Liu, G.; Shang, X.; Xiong, G.; et al. Cholesterol-Rich Dietary Pattern during Early Pregnancy and Genetic Variations of Cholesterol Metabolism Genes in Predicting Gestational Diabetes Mellitus: A Nested Case-Control Study. *The American Journal of Clinical Nutrition* **2023**, *118*, 966–976, doi:https://doi.org/10.1016/j.ajcnut.2023.08.017.
28. Shan, X.; Peng, C.; Zou, H.; Pan, Y.; Wu, M.; Xie, Q.; Lin, Q. Association of Vegetables-Fruits Dietary Patterns with Gestational Diabetes Mellitus: Mediating Effects of Gut Microbiota. *Nutrients* **2024**, *16*, 2300–2300, doi:https://doi.org/10.3390/nu16142300.
