# Supplementary material for: Are Maternal Dietary Patterns During Pregnancy Associated with the Risk of Gestational Diabetes Mellitus? A Systematic Review of Observational Studies
Source: Nutrients. 2024 Oct 25;16(21):3632. doi: 10.3390/nu16213632 (PMC11547687; doi:10.3390/nu16213632)
Supplement: Supplementary file 1 [file nutrients-16-03632-s001.zip › nutrients-3274816 suppl materials SIII.pdf]

Supplementary Material III

Table SIII.1. Overview of the dietary patterns and main findings on the links between maternal dietary patterns and gestational diabetes mellitus (*n*=28). Only the most adjusted models are presented. To facilitate data interpretation, statistically significant data are presented in bold letters. The orange colour was used to depict the detrimental effect, the green to illustrate the protective effect, and the light grey to represent the null effect of a specific DP.

Study	Dietary Patterns nominations described in the original publications	Main findings
**Cohort Studies**		
**Radesky et al., (2007)** [44]	• "Prudent" (NUT)<br>• "Western" (WES)	Prudent DP: High vs low adherence: (OR: 1.13, 95%CI: 0.59-2.16, *p*=0.35)<br>Western DP: High vs low adherence: (OR: 0.87, 95%CI: 0.41-1.83, *p*=0.80)
**He et al. (2015)** [45]	• "Vegetable" **(PLA)**<br>• "Sweets and seafood" **(WES)**<br>• "Protein-rich" (WES)<br>• "Prudent" (NUT)	Vegetable DP: T3 vs T1 (RR: 0.79, 95%CI: 0.64-0.97, ***p*=0.036**)<br>Sweets and seafood DP: T3 vs T1 (RR: 1.23, 95%CI: 1.02-1.49, ***p*=0.010**)<br>Protein-rich DP**:** T3 vs T1 (RR: 0.95, 95%CI: 0.78-1.16, *p*=0.659)<br>Prudent DP**:** T3 vs T1 (RR: 1.00, 95%CI: 0.82-1.22, *p*=0.914)<br>The protective effect of a high vegetable pattern score was more evident among women who had a family history of DM (***p*=0.022**)
**Tryggvadottir et al. (2015)** [46]	• "Prudent" **(NUT)**	Better adherence to the prudent DP was associated with a significant ↓ GDM risk (OR: 0.44, 95%CI: 0.21-0.90, ***p*≤0.05**).<br>The association between GDM and prudent DP remained even when the analysis was limited to OV/PB (*n*=82) (OR: 0.31, 95%CI: 0.13-0.75, ***p*≤0.05**)
**Nascimento et al. (2016)** [47]	• "Traditional" (NUT)<br>• "Mixed" (WES)<br>• "Western" (WES)	Traditional DP: T3 vs T1 (RR: 0.88, 95%CI: 0.49-1.58, *p*>0.05)<br>Mixed DP: T3 vs T1 (RR: 0.93, 95%CI: 0.51-1.71, *p*>0.05)<br>Western DP: T3 vs T1 (RR: 0.78, 95%CI: 0.43–1.43, *p*>0.05)
**Du et al., (2017)**[23]	• "Traditional" **(MIS)**<br>• "Mixed" (MIS)<br>• "Western" **(WES)**<br>• "Prudent" **(NUT)**	Traditional DP: Q3 vs Q1 (OR: 2.86, 95%CI: 1.19-6.83, ***p*=0.005**)<br>Q4 vs Q1 (OR: 2.92, 95%CI: 1.19-7.17, ***p*=0.005**)<br>Mixed DP: Q4 vs Q1 (OR: 0.70, 95%CI: 0.32-1.55, *p*=0.56)<br>Western DP: Q4 vs Q1 (OR: 1.68, 95%CI: 0.66-4.29, *p*=0.15)<br>Prudent DP: Q4 vs Q1 (OR: 0.49, 95%CI: 0.20-1.22, *p*=0.35)<br>Prudent (ref.) vs Western DP: OR: 4.40, 95%CI: 1.58-12.22, ***p*=0.004**<br>Prudent (ref.) vs traditional DP: OR: 4.88, 95%CI: 1.79-13.32, ***p*=0.002**<br>Prudent (ref.) vs mixed DP: OR: 1.61, 95%CI: 0.52-4.97, *p*=0.410
**Hajianfar et al. (2018)** [24]	• "Traditional" (MIS)<br>• "Western" (WES)<br>• Healthy (MIS)	Traditional DP: Q4 vs Q1 (OR: 1.71, 95%CI: 0.75-3.88, *p*=0.28)<br>Western DP: Q4 vs Q1 (OR: 0.52, 95%CI: 0.22-1.25, *p*=0.39)<br>Healthy DP: Q4 vs Q1 (OR: 1.04, 95%CI: 0.46-2.31, *p*=0.06)

**Mak et al. (2018)** [48]	• "Plant-based" (PLA)<br>• "Meat-based" (WES)<br>• "High protein-low starch" (NUT)	Plant-based DP: T3 vs T1 (OR: 0.97, 95%CI: 0.64-1.47, *p*=0.915)<br>Meat-based DP: T3 vs T1 (OR: 0.89, 95%CI: 0.58-1.36, *p*=0.534)<br>High protein-low starch DP: T3 vs T1 (OR: 0.73, 95%CI: 0.48-1.10, *p*=0.132)<br>In subgroup analyses, a statistically significant reduction in GDM risk was reported among overweight women in high protein-low starch DP (OR: 0.29, 95%CI: 0.09-0.94; ***p* =0.049**).
**Zhou et al. (2018)** [49]<br>*The "Tongji Maternal and Child Health Cohort"*	• "Beans–vegetables" (PLA)<br>• "Nuts–whole grains" (NUT)<br>• "Organs–poultry–seafood" (MIS)<br>• "Fish–meat–eggs" **(MIS)**<br>• "Rice–wheat–fruits" **(PLA)**	Beans–vegetables DP: Q4 vs Q1 (OR: 0.97, 95%CI: 0.64-1.46, *p*=0.649)<br>Nuts–whole grains DP: Q4 vs Q1 (OR: 1.25, 95%CI: 0.84-1.86, *p*=0.308)<br>Organs–poultry–seafood DP: Q4 vs Q1 (OR: 1.01, 95%CI: 0.68-1.51, *p*=0.918)<br>Fish–meat–eggs DP: Q4 vs Q1 (OR: 1.83, 95%CI: 1.21-2.79, ***p*=0.007**)<br>Rice–wheat–fruits DP: Q3 vs Q1 (OR:0.54, 95%CI: 0.36-0.83, ***p*=0.010**)<br>The "fish-meat-eggs" DP - GDM relation was no longer significant after further adjustment for total protein (energy %) (OR: 1.42, 95%CI: 0.90-2.22, *p*=0.162), or animal protein (energy %) (OR: 1.51, 95%CI: 0.94-2.44, *p*=0.119), or total carbohydrate (energy %) (OR: 1.48, 95%CI: 0.92-2.36, *p*=0.129), or ratio of protein: carbohydrate (OR: 1.35, 95%CI: 0.84-2.16, *p*=0.263). Stratification by the BMI, in women with BMI≥24.0 kg/m^2, the Q4 vs Q1 in the "fish–meat–eggs" DP was associated with ↑ GDM risk (OR: 2.84, 95%CI: 1.03-7.80, ***p*=0.021**).<br>Stratification by the family DM history indicated that the positive association between "Fish–meat–eggs" DP and the GDM risk was stronger.
**Hu et al. (2019)**<br>The "Born in Shenyang Cohort Study" [50]	• "Traditional" **(MIS)** ◊<br>• "Sweet foods" (WES) ◊<br>• "Fried food–beans" (MIS) ◊<br>• "Whole grain-seafood" **(NUT)** ◊<br>• "Traditional" **(PLA)** ∧<br>• "Fish–seafood" (NUT) ∧<br>• "Protein–sweets" (MIS) ∧	Traditional DP: Q4 vs Q1 (OR: 0.40, 95%CI: 0.23-0.71, ***p*=0.005**) ◊<br>Sweet foods DP: Q4 vs Q1 (OR: 0.73, 95%CI: 0.46, 1.16, *p*=0.199) ◊<br>Fried food–beans DP: Q4 vs Q1 (OR: 1.08, 95%CI: 0.67-1.74, *p*=0.940) ◊<br>Whole grain-seafood DP: Q4 vs Q1 (OR: 1.73, 95%CI: 1.10-2.74, ***p*=0.007**) ◊<br>Traditional DP: Q4 vs Q1 (OR: 0.44, 95%CI: 0.27-0.70, ***p*<0.001**) ∧<br>Fish–seafood DP: Q4 vs Q1 (OR: 0.95, 95%CI: 0.56-1.59, *p*=0.986) ∧<br>Protein-sweets DP: Q4 vs Q1 (OR: 1.18, 95%CI: 0.69-2.03, *p*=0.434) ∧<br>In subgroup analyses, the protective effect of traditional DP (3-d FD-FFQ) was more pronounced among women≥35 years old.

**Lawrence, Wall, & Bloomfield (2020) (26)**	• "Junk" **(WES)**<br>• "Health conscious" **(NUT)**<br>• "Traditional/White bread" **(WES)**<br>• "Fusion Protein" **(MIS)**	Junk DP: T3 vs T1 (OR: 0.49, 95%CI: 0.34-0.70, ***p*<0.0005**)<br>Health-conscious DP: T3 vs T1 (OR: 1.24, 95%CI: 0.87-1.77, *p*=0.244)<br>Traditional/White bread DP: T3 vs T1 (OR: 0.47, 95%CI: 0.32-0.68, ***p*<0.0005**)<br>Fusion Protein DP: T3 vs T1 (OR: 1.25, 95%CI: 0.87-1.81, *p*=0.231)<br>When stratified by the timing of GDM diagnosis, the inverse relationship between "Junk" and "Traditional/White bread" DP and GDM remained only for women diagnosed before the antenatal interview (***p*<0.0005**). In women diagnosed with GDM after the antenatal interview, only the association between the "Junk" DP and the likelihood of GDM diagnosis remained significant (***p*=0.036**).
**Yong et al. (2020)** [51]	• "DP4": vegetables, nuts, seeds & legumes, green leafy vegetables, fruits **(PLA)** †<br>• "DP5": condiments & spices, sugar, spread & creamer, oils & fats **(WES)** †<br>• "DP6": high protein, sugars, & energy **(WES)** †<br>• "DP7": other vegetables, nuts, seeds & legumes, green leafy vegetables, pasta **(PLA)** ¥<br>• "DP8": condiments & spices, sugar, spread & creamer **(WES)** ¥<br>• "DP9": high protein, sugars, energy, fruits **(WES)** ¥	DP4: High vs low adherence (OR: 0.81, 95%CI: 0.38–1.71, *p*=0.57) †<br>DP5: High vs low adherence (OR: 0.28, 95%CI: 0.11–0.68, ***p*=0.01**) †<br>DP6: High vs low adherence (OR: 1.15, 95%CI: 0.54–2.47, *p*=0.72) †<br>DP7: High vs low adherence (OR: 0.51, 95%CI: 0.24–1.11, *p=0.07*) ¥<br>DP8: High vs low adherence (OR: 0.73, 95%CI: 0.34–1.58, *p*=0.42) ¥<br>DP9: High vs low adherence (OR: 0.73, 95%CI: 0.33–1.63, *p*=0.44) ¥<br>When stratified, the association between DP5 and GDM was only observed among UN/NW women (OR: 0.27, 95%CI: 0.08–0.87**, *p*=0.02**).
**De Seymour et al. (2022)** [52]	• "Fish, poultry and vegetables (FPV)-based" **(NUT)**<br>• "Pasta, sweetened beverages, oils, and condiments (PSO)-based" **(WES)**	FPV-based DP: High vs low adherence (OR: 1.03, 95%CI: 0.88-1.21, *p*=0.689)<br>PSO-based DP: High vs low adherence (OR: 0.96, 0.82-1.13, *p*=0.636)
**Wang et al. (2023)** [53]	• "Tuber crops-fish" **(MIS)** ?<br>• "Legumes-vegetables-fruits" **(PLA)** ?<br>• "Meats-eggs-dairy" **(WES)** ?<br>• "Fruits-eggs-leafy vegetable" **(MIS)** ¤<br>• "Eggs-fish" **(MIS)** ¤<br>• "Root vegetables-fruits" **(PLA)** ¤	Tuber crops-fish DP: Q1 vs Q4 (OR: 1.13, 95%CI: 0.80-1.58, *p*=0.688) ?<br>Legumes-vegetables-fruits DP: Q1 vs Q4 (OR: 0.88, 95%CI: 0.60-1.31, *p*=0.670) ?<br>Meats-eggs-dairy DP: Q1 vs Q4 (OR: 0.80, 95%CI: 0.54-1.20, *p*=0.316) ?<br>Fruits-eggs-leafy vegetable DP: Q1 vs Q4 (OR: 0.81, 95%CI: 0.48-1.38, *p*=0.437) ¤<br>Eggs-fish DP: Q1 vs Q4 (OR: 0.89, 95%CI: 0.63-1.26, *p*=0.649) ¤<br>Root vegetables-fruits DP: Q1 vs Q4 (OR: 1.01, 95%CI: 0.70-1.45, *p*=0.710) ¤
**Cross-Sectional Study**		

**Shin et al. (2015)** [25]	• "High refined grains, fats, oils, and fruit juice" **(WES)**<br>• "High nuts, seeds, solid fat, and soybean; low milk and cheese" **(MIS)**<br>• "High added sugar and organ meats, low fruits, vegetables, and seafood" **(WES)**	"High refined grains, fats, oils and fruit juice" DP: T3 vs T1 (OR: 4.9, 95%CI: 1.4–17.0, ***p*=0.007**).<br>"High nuts, seeds, solid fat and soybean, low milk and cheese" DP: T3 vs T1 (OR: 7.5, 95%CI: 1.8–32.3, ***p*=0.009**)<br>"High added sugar and organ meats, low fruits, vegetables and seafood" DP: T3 vs T1 (OR: 22.3, 95%CI: 3.9–127.4, ***p*<0.0001**)
**De Seymour et al. (2016)** [54]	• "Vegetable-fruit-rice-based-diet" **(NUT)**<br>• "Seafood-noodle-based-diet" **(MIS)**<br>• "Pasta-cheese-processed-meat-diet" **(WES)**	"Vegetable-fruit-rice-based-diet" DP: Higher score (OR: 1.10, 95%CI: 0.90-1.35, *p*=0.36)<br>"Seafood-noodle-based-diet" DP: Higher score (OR: 0.74, 95%CI: 0.59-0.93, ***p*<0.01**)<br>"Pasta-cheese-processed-meat-diet" DP: Higher score (OR: 0.96, 95%CI: 0.79-1.17, *p*=0.71)<br>When stratified by ethnicity, a weak association between higher score in Vegetable-fruit-rice-based-diet" DP and GDM risk (OR: 0.75, 95%CI: 0.56-1.00, ***p*=0.047**) was reported in Chinese women (*n*=505). When stratified by previous history of GDM, the effect of "seafood-noodle-based-diet" DP on GDM remained statistically significant only for women without previous GDM history (OR: 0.70, 95%CI: 0.55-0.88, ***p*=0.002**).
**Flynn et al. (2016)** [22]	• "Fruit and vegetables" **(PLA)**<br>• "African/Caribbean" **(WES)**<br>• "Processed" **(WES)**<br>• "Snacks" **(WES)**	Fruit and vegetables DP: Q4 vs Q1 (OR: 1.03, 95%CI: 0.64-1.68, *p*=0.891)<br>African/Caribbean DP: Q4 vs Q1 (OR: 2.46, 95%CI: 1.41-4.31, *p*=**0.010**)<br>Processed: Q4 vs Q1 (OR: 2.05, 95%CI: 1.23-3.41, ***p*=0.022**)<br>Snacks: Q4 vs Q1 (OR: 1.24, 95%CI: 0.76-2.01, *p*=0.666)
**Sartorelli et al. (2019)** [55]	• "DP1": high rice, beans, and vegetables, with low full-fat dairy products, biscuits, and sweets **(PLA)**<br>• "DP2": high red meats, full-fat dairy products, chocolate powder and fruits, with low chicken and margarine **(WES)**	DP1: T3 vs T1 (OR: 0.58, 95%CI: 0.36-0.95, ***p*=0.03**)<br>DP2: T3 vs T1 (OR: 1.48, 95%CI: 0.91-2.40, *p*=0.11)
**Zuccolotto et al. (2019)** [7]	• "Traditional Brazilian" **(MIS)**<br>• "Snacks" **(WES)**<br>• "Coffee" (-)<br>• "Healthy" **(PLA)**	Traditional Brazilian DP: T3 vs T1 (OR: 0.64, 95%CI: 0.39–0.99, *p*=0.06)<br>Snacks DP: T3 vs T1 (OR:0.96, 95%CI: 0.59–1.55, *p*=0.88)<br>Coffee DP: T3 vs T1 (OR: 0.97, 95%CI: 0.59–1.59, *p*=0.92)<br>Healthy DP: T3 vs T1 (OR: 1.04, 95%CI: 0.64–1.68, *p*=0.87)
**Pajunen et al. (2022)** [56]	• "Healthier" **(NUT)**<br>• "Unhealthier" **(WES)**	Healthier DP: $Q_{1/5th}4$ vs $Q_{1/5th}1$ (OR: 0.27, 95%CI: 0.11–0.70, ***p*=0.007**),<br>$Q_{1/5th}5$ vs $Q_{1/5th}1$ (OR: 0.82, 95%CI: 0.38–1.74), *p*=0.602)<br>Unhealthier DP: $Q_{1/5th}5$ vs $Q_{1/5th}1$ (OR: 1.04, 95%CI: 0.48–2.22, *p*=0.928).

**Wu et al. (2022)** [57]	• "White meat" **(MIS)**<br>• "Red meat" **(WES)**<br>• "Plant–dairy–eggs" **(NUT)**	Plant–dairy–eggs *(ref.)* vs white meat DP: OR: 1.83, 95%CI: 1.04–3.24, ***p*<0.05**<br>Plant–dairy–eggs *(ref.)* vs red meat DP: OR: 1.80, 95%CI: 1.06–3.07, ***p*<0.05**
**Ebrahimi et al. (2024)** [58]	• "First" **(WES)**<br>• "Second" (WES)<br>• "Third" (NUT)	First DP: (Early pregnancy: OR: 0.99, 95%CI: 0.98-1.00, *p*=0.120; Late pregnancy: OR: 0.99, 95%CI: 0.98-0.99, ***p*=0.023**)<br>Second DP: (Early pregnancy: OR: 0.99, 95%CI: 0.99-1.00, *p*=0.585; Late pregnancy: OR: 0.99, 95%CI: 0.99-1.00, *p*=0.438)<br>Third DP: (Early pregnancy: OR: 1.00, 95%CI: 0.99-1.01, *p*=0.763; Late pregnancy: OR: 1.00, 95%CI: 0.99-1.01, *p*=0.775)
**Case–Control**		
**Zareei et al. (2018)** [59]	• "Healthy" **(MIS)**<br>• "Unhealthy" **(WES)**	Healthy DP: Q4 vs Q1 (OR: 0.28, 95%CI: 0.096–0.84, ***p*=0.023**)<br>Unhealthy DP: Q4 vs Q1 (OR: 2.84, 95%CI: 1.04-7.75, ***p*=0.042**)
**Chen et al. (2020)** [8]	• "Vegetables" **(NUT)**<br>• "Cereals" (PLA)<br>• "Meats" (MIS)	Vegetables DP: Q4 vs Q1 (1st trimester: OR: 0.78, 95%CI: 0.65–0.91, *p*-value=0.017, 2nd trimester: OR: 0.74, 95%CI: 0.63–0.87, ***p*=0.001**)<br>Cereals DP: Q4 vs Q1 (OR: N/A, *p*>0.05)<br>Meats DP: Q4 vs Q1 (OR: N/A, *p* >0.05)<br>After stratification by BMI and maternal age, the statistically significant associations between the vegetables DP and GDM risk were only found in women with BMI<24kg/m^2 (***p*=0.015** and ***p*=0.002** for the 1st and 2nd trimester, respectively) and <35 years old (***p*=0.001** and ***p*<0.001** for the 1st and 2nd trimester, respectively).
**Roustazadeh et al. (2021)** [60]	• "Fruits and dairy products" **(NUT)**<br>• "Red meat and plant-based foods" (NUT)<br>• "Snacks and high-fat foods" (WES)<br>• "Carbohydrate-rich foods" (MIS)	Fruits and dairy products DP: Q4 vs Q1 (OR: 0.50, 95%CI: 0.28-0.88, ***p*=0.019**)<br>Red meat and plant-based foods DP: Q4 vs Q1 (OR: 0.66, 95%CI: 0.37-1.17, *p*=0.950)<br>Snacks and high-fat foods DP: Q4 vs Q1 (OR: 0.93, 95%CI: 0.50-1.74, *p*=0.672)<br>Carbohydrate-rich foods DP: Q4 vs Q1 (OR: 1.79, 95%CI: 0.98-3.28, *p*=0.559).
**Waheby et al. (2021)** [61]	• "Healthy" **(NUT)**<br>• "Unhealthy" **(WES)**	Healthy DP: T3 vs T1 (OR: 0.73, 95%CI: 0.60-0.90, ***p*=0.002**)<br>Unhealthy DP: T3 vs T1 (OR: 3.41, 95%CI: 0.03-0.15, ***p*=0.003**)
**Liu et al., (2022)** [62]	• "DP1" (MIS)<br>• "DP2" **(WES)**<br>• "DP3" (- *)	DP1: Q4 vs Q1 (OR: 0.48, 95%CI: 0.14–1.68, *p*=0.450)<br>DP2: Q4 vs Q1 (OR: 2.96, 95%CI: 0.94–9.36, ***p*=0.004**)<br>DP3: N/A
**Cui et al. (2023)** [63]	• "Cholesterol-rich" **(WES)**	The GDM risk was elevated with increased DP scores (***p*=0.010**). This relationship was more pronounced among women having a higher GRS.<br>Q1 vs Q2 (OR: 1.61, 95%CI: 1.05-2.46, ***p*=0.030**)<br>Q1 vs Q3 (OR: 1.59, 95%CI: 1.04-2.43, ***p*=0.034**)<br>Q1 vs Q4 (OR: 1.92, 95%CI: 1.23-2.99, ***p*=0.004**)

**Shah et al. (2024)** [64]	• "Processed food" (WES)<br>• "Meat" (MIS)<br>• "Fungi and algae-beans" (PLA)<br>• "Cereals and potatoes-eggs and milk" (NUT)<br>• "Vegetables-fruits" **(NUT)**	Higher factor scores (≥ 0.07) in the "Vegetables-Fruits" DP were associated with a reduced risk of GDM compared to those with factor scores below 0.07 (OR: 0.33; 95% CI: 0.15-0.74, ***p*=0.008**). For the other DPs, comparisons between cases and controls showed no statistically significant differences between high and low adherence.

◊ 3-d FD; ∧ FFQ; † First trimester; ¥ Second trimester; ⁇ PCA; ¤ RRR; (-) No classification; * The explained variation of DP3 was relatively small and thus was not further analysed in the original article. BMI: Body Mass Index; DM: Diabetes Mellitus; DP: Dietary Pattern; FFQ: Food Frequency Questionnaire; GDM: Gestational Diabetes Mellitus; GRS: Genetic Risk Score; GWG: Gestational Weight Gain; FD: Food Records; MIS: Miscellaneous Dietary Pattern; n=Number; N/A: non-available; NUT: Nutritious Dietary Pattern; OR: Odd Ratio; OV/OB: Overweight/obese; PA: Physical Activity; PCA: Principal Component Analysis; PLA: Plant-based Dietary Pattern; Q: Quartile; $Q_{1/5th}$: Quintile; ref.: reference; RR: Relative Risk; RRR: Reduced Rank Correlation; T: Tertile; UN/NW: Underweight/normal weight, WES: Westernized Dietary Pattern.

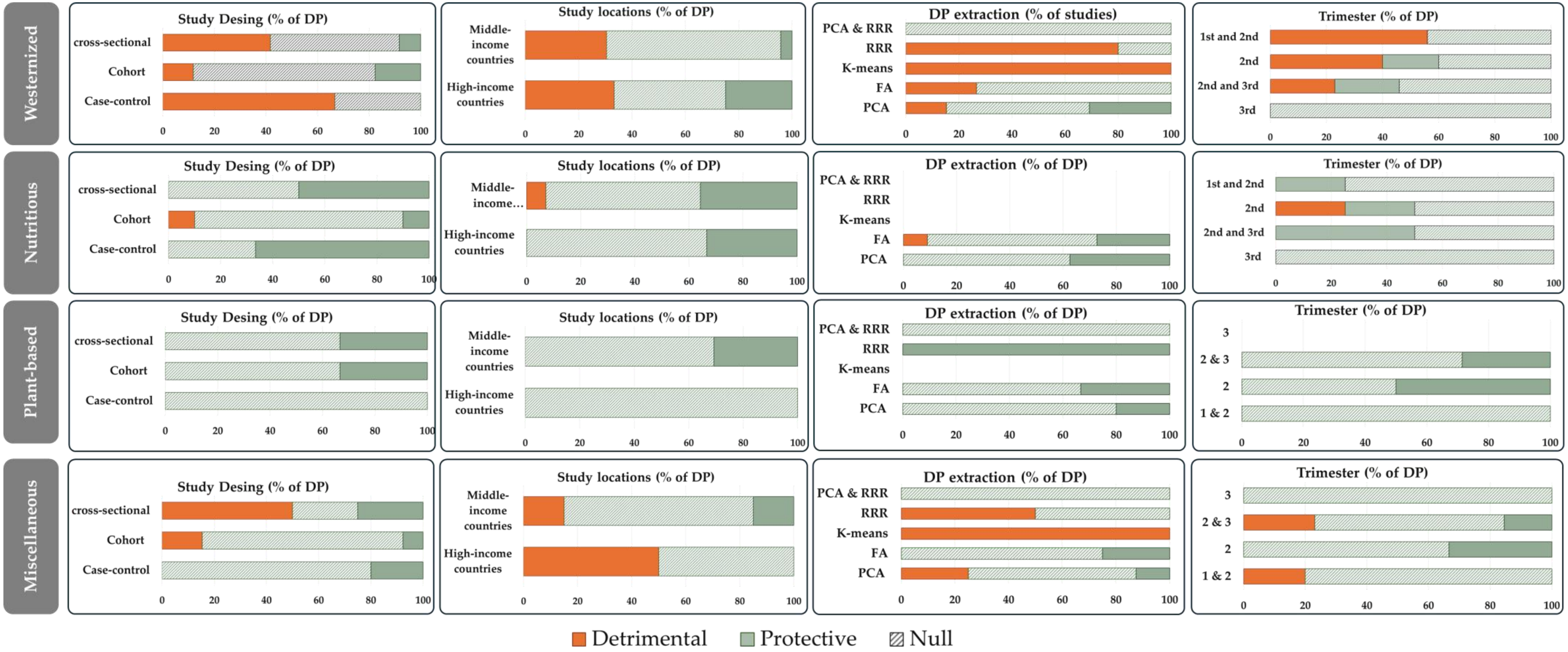

Figure SIII.1 Associations between dietary patterns (DP) and risk of gestational diabetes mellitus. The findings within each DP category, expressed as a percentage of DP, are stratified by the study design and country, dietary pattern extraction methods, and trimester during which dietary assessments were carried out. FA: factor analysis; PCA: Principal Component Analysis; RRR: Reduced Rank Regression.

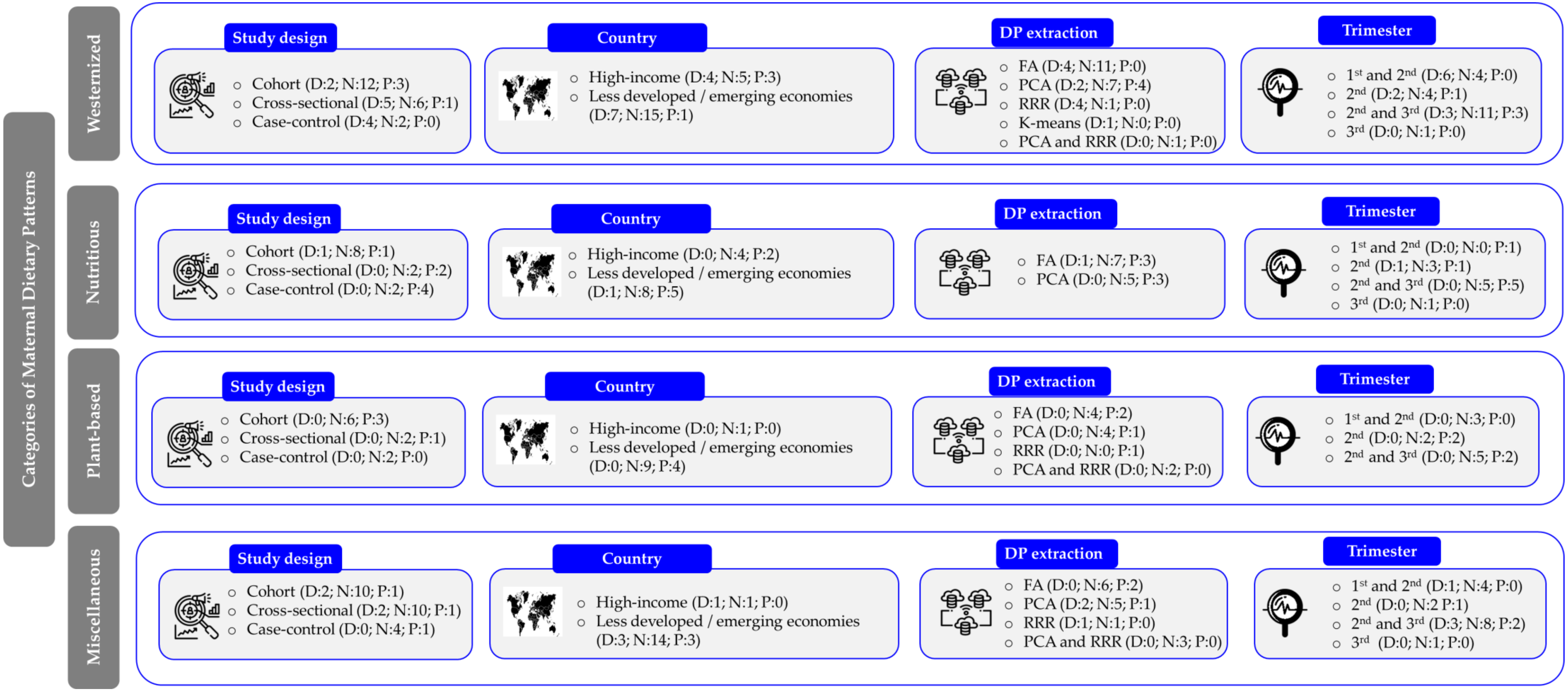

Figure SIII.2 Associations between maternal dietary patterns, during pregnancy, and risk of gestational diabetes mellitus. The findings within each dietary pattern category are stratified by the study design and country, dietary pattern extraction methods, and trimester during which dietary assessments were carried out. Withing the parenthesis are given the number of dietary patterns with Detrimental (D); Null (N), and Protective (P) associations. FA: factor analysis; PCA: Principal Component Analysis; RRR: Reduced Rank Regression.
