# Supplementary material for: Are Maternal Dietary Patterns During Pregnancy Associated with the Risk of Gestational Diabetes Mellitus? A Systematic Review of Observational Studies
Source: Nutrients. 2024 Oct 25;16(21):3632. doi: 10.3390/nu16213632 (PMC11547687; doi:10.3390/nu16213632)
Supplement: Supplementary file 1 [file nutrients-16-03632-s001.zip › nutrients-3274816 suppl materials SI.pdf]

Supplementary Material I

Table SI-1. Newcastle - Ottawa Quality Assessment Scale for Prospective Cohort Studies [1].

	**1. Selection**
**I.**	**Representativeness of the exposed cohort:**
a)	truly representative of the average pregnant women in the community. ★
b)	somewhat representative of the average pregnant women in the community. ★
c)	selected group of users e.g. volunteers, certain group of women.
d)	no description of the derivation of the cohort.
**II.**	**Selection of the non-exposed cohort:**
a)	drawn from the same community as the exposed cohort. ★
b)	drawn from a different source.
c)	no description of the derivation of the non-exposed cohort.
**III.**	**Ascertainment of exposure (dietary patterns):**
a)	secure record (e.g. validated Food Frequency Questionnaires). ★
b)	structured interview. ★
c)	written self-report.
d)	no description.
**IV.**	**Demonstration that outcome of interest (Gestational Diabetes Mellitus) was not present at start of study:**
a)	yes ★
b)	no
	**2. Comparability**
**V.**	**Comparability of cohorts on the basis of the design or analysis:**
a)	study controls for maternal age and Body Mass Index. ★
b)	study controls for any additional factor (e.g. history of Gestational Diabetes Mellitus or family history of diabetes mellitus, ethnicity, physical activity etc.). ★
	**3. Outcome**
**VI.**	**Assessment of outcome:**
a)	independent blind assessment. ★
b)	record linkage. ★
c)	self-report.
d)	no description.
**VII.**	**Was follow-up long enough for outcomes to occur:**
a)	yes (e.g. till diagnosis) ★
b)	no
**VIII.**	**Adequacy of follow up of cohorts:**
a)	complete follow up - all subjects accounted for. ★
b)	subjects lost to follow up unlikely to introduce bias (small number lost ≤ adequate %) or description provided of those lost. ★
c)	follow up rate < 70% and no description of those lost.

d)	no statement.

Table SI-2. Newcastle - Ottawa Quality Assessment Scale for Cross-sectional Studies [2]

	**1. Selection**
**I.**	**Representativeness of the sample:**
a)	truly representative of the average in the target population. (all subjects or random sampling) ★
b)	somewhat representative of the average in the target population. (non- random sampling) ★
c)	selected group of users.
d)	no description of the sampling strategy.
**II.**	**Sample size:**
a)	justified and satisfactory. ★
b)	not justified.
**III.**	**Non-respondents:**
a)	comparability between respondents and non-respondents characteristics is established, and the response rate is satisfactory. ★
b)	the response rate is unsatisfactory, or the comparability between respondents and non-respondents is unsatisfactory.
c)	No description of the response rate or the characteristics of the responders and the non-responders.
**IV.**	**Ascertainment of the exposure (risk factor):**
a)	validated measurement tool. ★★
b)	non-validated measurement tool, but the tool is available or described. ★
c)	no description of the measurement tool.
	**2. Comparability**
**V.**	**The subjects in different outcome groups are comparable, based on the study design or analysis. Confounding factors are controlled:**
a)	study controls for maternal age and Body Mass Index. ★
b)	study controls for any additional factor (e.g. history of Gestational Diabetes Mellitus or family history of diabetes mellitus, ethnicity, physical activity etc.). ★
	**3. Outcome**
**VI.**	**Assessment of the outcome:**
a)	independent blind assessment. ★★
a)	record linkage. ★★
b)	self-report. ★
c)	no description.
**VII.**	**Statistical test:**
a)	The statistical test used to analyze the data is clearly described and appropriate, and the measurement of the association is presented, including confidence intervals and the probability level (*p*-value). ★
b)	The statistical test is not appropriate, not described, or incomplete.

Table SI-3. Newcastle - Ottawa Quality Assessment Scale for Case-control studies [1].

	**1. Selection**
**I.**	**Is the case definition adequate:**
a)	yes, with independent validation (e.g. hospital records). ★
b)	yes, e.g. record linkage or based on self-reports.
c)	no description.
**II.**	**Representativeness of the cases:**
a)	consecutive or obviously representative series of cases. ★
b)	potential for selection biases or not stated.
**III.**	**Selection of controls:**
a)	community controls. ★
b)	hospital controls.
c)	no description.
	**2. Comparability**
**IV.**	**Comparability of cases and controls on the basis of the design or analysis:**
a)	study controls for maternal age and Body Mass Index. ★
b)	study controls for any additional factor (e.g. history of Gestational Diabetes Mellitus or family history of diabetes mellitus, ethnicity, physical activity etc.). ★
	**3. Exposure**
**V.**	**Assessment of exposure:**
a)	secure records or biochemical analysis. ★
b)	structured interview blind to case/control status. ★
c)	interview not blinded to case/control status.
d)	written self-report or medical records only
e)	no description.
**VI.**	**Same method of ascertainment for cases and controls:**
a)	yes ★
b)	no
**VII.**	**Non-response rate:**
a)	Same rate for both groups. ★
b)	Non respondents described.
c)	rate different for both groups.

References

1. Wells, G.; Shea, B.; O'Connell, D.; Peterson, J.; Welch, V.; Losos, M.; Tugwell, P. The Newcastle-Ottawa Scale (NOS) for Assessing the Quality of Nonrandomised Studies in Meta-Analyses Available online: https://www.ohri.ca/programs/clinical_epidemiology/oxford.asp.
2. Modesti, P.A.; Reboldi, G.; Cappuccio, F.P.; Agyemang, C.; Remuzzi, G.; Rapi, S.; Perruolo, E.; Parati, G. Panethnic Differences in Blood Pressure in Europe: A Systematic Review and Meta-Analysis. PLOS ONE 2016, 11, e0147601, doi:https://doi.org/10.1371/journal.pone.0147601.
